# Transcriptomic Reprograming of *Xanthomonas campestris* pv. *campestris* after Treatment with Hydrolytic Products Derived from Glucosinolates

**DOI:** 10.3390/plants10081656

**Published:** 2021-08-11

**Authors:** Pari Madloo, Margarita Lema, Victor Manuel Rodríguez, Pilar Soengas

**Affiliations:** 1 Group of Genetics, Breeding and Biochemistry of Brassicas, Misión Biológica de Galicia, Spanish Council for Scientific Research (MBG-CSIC), 36143 Pontevedra, Spain; paribrokanloui.madloo@rai.usc.es (P.M.); vmrodriguez@mbg.csic.es (V.M.R.); 2 Department of Functional Biology, School of Biology, Universidade of Santiago de Compostela, 15782 Santiago de Compostela, Spain; margarita.lema@usc.es

**Keywords:** black rot, plant–pathogen interaction, plant secondary metabolites, isothiocyanate, Brassicaceae

## Abstract

The bacterium *Xanthomonas campestris* pv. *campestris* (*Xcc*) causes black rot disease in *Brassica* crops. Glucosinolates are known to be part of the defence system of *Brassica* crops against *Xcc* infection. They are activated upon pathogen attack by myrosinase enzymes. Their hydrolytic products (GHPs) inhibit the growth of *Xcc* in vitro. However, the mechanisms underlying this inhibition and the way *Xcc* can overcome it are not well understood. We studied the transcriptomic reprogramming of *Xcc* after being supplemented with two chemically different GHPs, one aliphatic isothiocyanate (allyl-ITC) and one indole (indol-3-carbinol), by RNA-seq. Based on our results, the arrest in *Xcc* growth is related to the need to stop cell division to repair damaged DNA and cell envelope components. Otherwise, GHPs modify energy metabolism by inhibiting aerobic respiration and increasing the synthesis of glycogen. *Xcc* induces detoxification mechanisms such as the antioxidant defence system and the multidrug efflux system to cope with the toxic effects driven by GHPs. This is the first time that the transcriptomic reprogramming of a plant pathogenic bacterium treated with GHPs has been studied. This information will allow a better understanding of the interaction of a plant pathogen mediated by GSLs.

## 1. Introduction

*Xanthomonas campestris* pv. *campestris* (*Xcc*) is a Gram-negative, aerobic, vascular, and motile bacterium with a single flagellum, which causes the disease identified as black rot in *Brassica* crops [1]. *Xcc* enters in the plant through the stomas, the hydathodes, and wounds. Once inside, the pathogen travels through the vascular system, invading the xylem and colonising the mesophyll. Disease symptoms are developed in the host plants in warm and humid conditions [2]. The infection is characteristic because it causes V-shaped necrotic lesions in the edges of leaves and necrosis and darkening of the veins of the leaves and the vascular tissue of the stem. As the disease progresses, wilting and necrosis throughout the plant occur [3]. Eleven races of *Xcc* have been recognised so far, defined based on their interaction with different *Brassica* cultivars following a gene-for-gene model [4,5,6]. Races 1 and 4 are the most pathogenic and widespread, accounting for 90% of the black rot in the world [4].

After the recognition of *Xcc*, the immune system of *Brassica* plants triggers various layers of defence, such as the synthesis of PR proteins and RLK receptors, the induction of defence hormones, an antioxidant response, and the synthesis of the secondary metabolites, glucosinolates (GSLs) [7]. GSLs are secondary metabolites found exclusively in Brassicaceae plants. Resistance to *Xcc* is related to the amount of GSLs in plants. The induction of the synthesis of GSLs confers a resistance to *Xcc* in *Brassica oleracea* [8]. A relationship was discovered [9] between the disease severity caused by *Xcc* and the GSL content in the Brassicaceae species cress (*Lepidium sativum*), salad rocket (*Eruca sativa*), and broccoli (*B. olerecea* L. var. *italica*). The modification of the content of specific GSLs affects the resistance to *Xcc*. After testing the genotypes of *B. oleracea* with a high and low content of the GSLs sinigrin, glucoiberin, and glucobrassicin, glucobrassicin clearly diminished the spread of *Xcc* in leaves; on the contrary, sinigrin and glucoiberin produced no clear effect [10].

GSLs are stored inactive in vacuoles. After tissue breakdown caused by injuries, pests, or necrotrophic pathogens, the glucosidases termed myrosinases convert GSLs into toxic products [11]. The toxicity significantly depends on the chemical structure. This ultimately relies on the structure of the original GSL and the presence of specific proteins in the host. Among the toxic products derived from GSLs, isothiocyanates (ITCs) are produced by default. The generation of nitriles and epithionitriles needs the presence of epithiospecific proteins, which modulate the activity of myrosinases [12]. GSL hydrolytic products (GHPs) have antimicrobial activities in vitro against plant pathogens [11,12,13]. Among them, ITCs are the most toxic, even at low concentrations [14]. Different evidence in vitro supports the antimicrobial activity of GHPs on *Xcc* growth [13,15,16]. The inhibition of growth is dependent on the concentration of GHP and on the race under study [13].

The concentration of GSL in *B. oleracea* modulates disease resistance to *Xcc* in plants [10]. GHPs can inhibit the growth of *Xcc* cultures [13]. The mechanisms through which GHPs exert their toxicity and the methods through which *Xcc* can overcome them are not quite understood. It has been effectively established that ITCs inhibit the growth of bacteria. Other effects include the inhibition of quorum sensing and the prevention of the formation of biofilms in the species *Escherichia coli*, *Pseudomonas aeruginosa*, and *Listeria monocytogenes* [17,18], as well as the induction of the stringent response [19] and of the oxidative stress response in *Campylobacter jejuni* [20]. ITCs can affect bacterial cell membrane integrity in a dose-dependent manner [21,22]. All these effects depend on the chemical structure of the ITC and on the bacteria under study. Apart from the inhibition of growth, information on the effects of other GHPs different from ITCs on bacteria is scarce. The effect of ITCs employing bacteria that are pathogenic for humans has been studied. Considering the prominent role of GSLs in plant defence, the response of plant pathogens under exposure to GHPs should be considered. Therefore, we aimed to study the reprogramming of *Xcc* race 1 transcriptome after exposure to two chemically different GHPs, one aliphatic ITC and one indole to provide further insights into the role of GHPs in the immune system of plants by considering the response of an important plant pathogen of *Brassica* crops.

## 2. Results

### 2.1. Bacterial Growth

**Figure 1:**
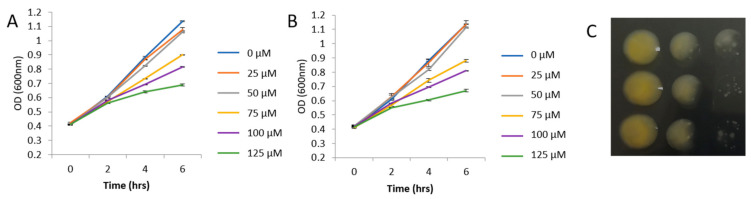
Growth of *Xanthomonas campestris* pv. *campestris* (*Xcc*) in a LB medium supplemented with different concentrations of (**A**) AITC and (**B**) I3C, monitored during the logarithmic phase for 6 h. (**C**) From top to bottom, 1/10 dilution series of *Xcc* supplemented with no GHP, AITC (100 µM), and I3C (100 µM) grown in LBA medium.

The growth of bacteria supplemented with different concentrations of GHPs was monitored during the log phase. The differences between the GHP-treated samples and the controls were significant at 4 and 6 h (Figure 1A,B). Both GHPs inhibited the growth of *Xcc* in a similar manner. A sublethal concentration of 100 μM was selected for further experiments.

### 2.2. GO Analysis

To provide a general overview of the results, a GO analysis with all the annotated transcripts was conducted for each comparison (GHPs vs. the control and AITC vs. I3C) with GO-Tool2. We provide a description of the most important features found in the category of biological process (BP). An analysis of the whole transcripts resulted in 147 significantly enriched GO terms (*p* < 0.05 and −1 < log_2_ fold change > 1), from which 11 were induced and 136 were repressed in the comparison AITC vs. the control. The most significantly induced terms were the energy reserve metabolic process (GO:0006112), the glycogen metabolic process (GO:0005977), DNA-strand elongation (GO:0022616), DNA ligation involved in DNA repair (GO:0051103), tRNA methylation (GO:0030488), the fructose 6-phosphate metabolic process (GO:0006002), and protein transport by the Sec complex (GO:0043952). Among the most significantly repressed GO terms were those related to the urea cycle (for example, GO:0000050), ATP hydrolysis coupled proton transport (GO:0015991) and the purine and pyrimidine nucleotide biosynthetic process (GO:0009174, GO:0009145). Other significant terms were related to the small molecule metabolic process (GO:0044281), aerobic respiration (GO:0009060), gluconeogenesis (GO:0006094), and the hexose metabolic process (GO:0006094, GO:0019319).

The GO analysis of I3C vs. the control produced 209 significant GO terms, of which 206 were repressed and 3 induced. The induced GO terms included sulphate transport (GO:0008272), sulphate transmembrane transport (GO:1902358), and protein transport by the Sec complex (GO:0043952). The list of repressed terms is similar to that detected in the comparison AITC vs. the control. In the comparison of AITC vs. I3C, all GO terms (173) displayed a higher expression in *Xcc* treated with AITC than with I3C. Among them, we observed terms related to purine and pyrimidine synthesis, aerobic respiration, the urea cycle, or ATP hydrolysis.

### 2.3. Differentially Expressed Genes

**Figure 2:**
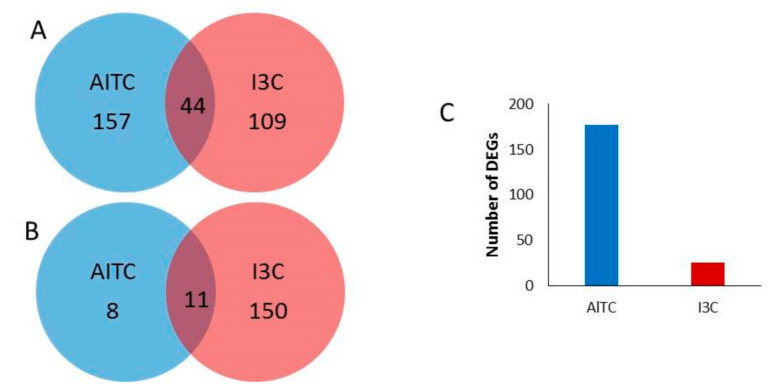
Venn diagrams showing overlapping (**A**) upregulated differentially expressed genes (DEGs) and (**B**) downregulated DEGs between the comparisons of AITC vs. the control and I3C vs. the control. (**C**) Number of upregulated DEGs by AITC or I3C in the comparison of AITC vs. I3C. DEGs were identified based on a false discovery rate ≤ 0.05 and −1 ≤ log_2_ fold change ≥ 1.

The number of DEGs was lower in response to AITC than in response to I3C compared to the control (220 vs. 314, respectively; Supplementary Tables S1 and S2). The majority of the DEGs in the I3C vs. the control treatment were downregulated. Forty-four genes were commonly induced by both treatments and eleven were repressed (Figure 2A,B). In the comparison of AITC vs. I3C, 177 DEGs were induced in response to AITC and 26 were induced in response to I3C (Figure 2C).

**Figure 3:**
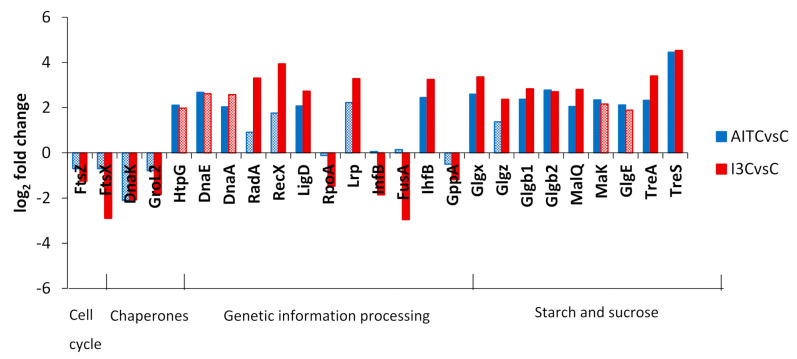
Log_2_ fold change of DEGs classified into KEGG categories of cell cycle, chaperones, genetic information processing, and starch and sucrose metabolism in the comparisons of AITC vs. the control and I3C vs. the control. Solid colours of the bars indicate a false discovery rate ≤ 0.05. A description of DEGs is provided in Supplementary Tables S1 and S2.

**Figure 4:**
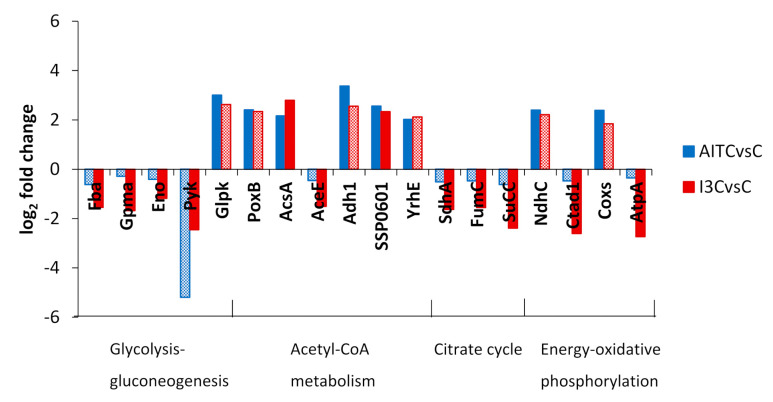
Log_2_ fold change of DEGs classified into KEGG categories of glycolysis-gluconeogenesis, acetyl-CoA metabolism, citrate cycle and energy-oxidative phosphorylation in the comparisons AITC vs. the control and I3C vs. the control. Solid colours of the bars indicate a false discovery rate ≤ 0.05. A description of DEGs is provided in Supplementary Tables S1 and S2.

**Figure 5:**
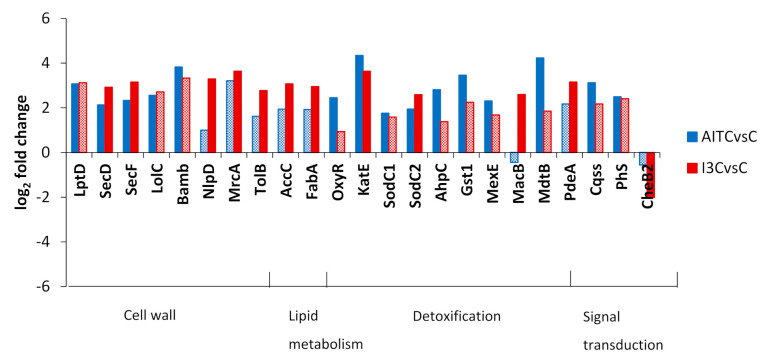
Log_2_ fold change of DEGs classified into KEGG categories of cell wall, lipid metabolism, detoxification, and signal transduction in the comparisons of AITC vs. the control and I3C vs. the control. Solid colours of the bars indicate a false discovery rate ≤ 0.05. A description of DEGs is provided in Supplementary Tables S1 and S2.

DEGs were classified into KEGG categories to better describe their function. The log_2_ fold change of DEGs cited in the text is shown in Figure 3, Figure 4 and Figure 5 for the comparisons of AITC vs. the control and I3C vs. the control. In Figure 3, we show the DEGs related to the cell cycle, chaperones, and genetic information processing. DEGs ftsX and ftsZ, which control the cell cycle, were repressed by I3C. DnaK was repressed by both GHPs. Several DEGs related to DNA repair were induced by both GHPs. Translation factors rpoA, infB, and fusA were repressed by I3C (Figure 3).

DEGs glgx, glgz, glgb1, glgb2 malQ, maK, and glgE controlling the synthesis of glucans were induced by one or both GHPs (Figure 3). TreA and treS were induced by both GHPs and they are related to the metabolism of trehalose (Figure 3).

I3C treatment inhibited DEGs related to glycolysis, the TCA cycle, and oxidative phosphorylation (Figure 4). Both treatments induced DEGs controlling alcohol dehydrogenases. AITC induced DEGs related to the synthesis of acetyl-CoA and its conversion into CO_2_ by formate dehydrogenases (Figure 4).

Both treatments induced DEGs controlling the synthesis of the cell envelope (Figure 5) including transporters of lipids and proteins. Multidrug transporters and antioxidant enzyme-related DEGs were induced by both GHPs (Figure 5). I3C induced the signal transduction factors cqss and phs as well as pdeA and repressed cheB2 (Figure 5).

**Figure 6:**
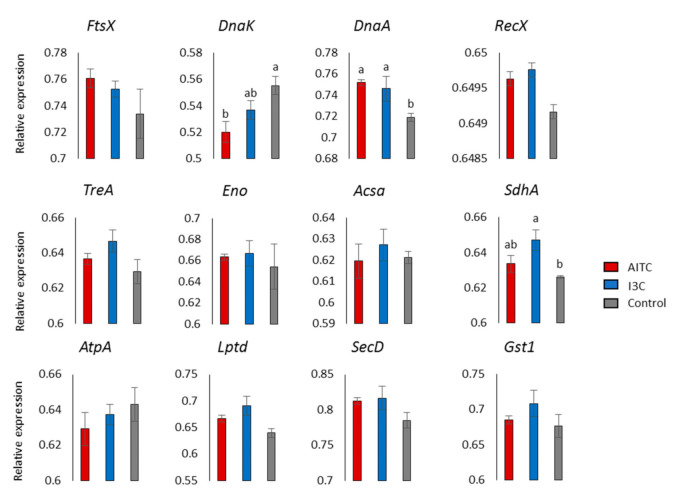
Relative expression patterns of twelve genes from *Xanthomonas campestris* pv. *campestris* treated with glucosinolate hydrolysis products and the control. Data are the average of three biological replicates ± SE. Letters at the top of the bars represent significant differences among treatments at *p*-value < 0.05.

Primers were designed to verify twelve DEGs using RT-qPCR (Supplementary Table S3). Only three DEGs presented significant differences among treatments (Figure 6). Generally speaking, the results are in good accordance with the expectations from the RNA-seq data (Figure 6), unless for DEGs ftsX, eno and sdha. Therefore, discussion of RNA-seq results related to these DEGs should be viewed with caution.

The growth of *Xcc* was arrested in the log phase after exposure to AITC and I3C in a dose-dependent manner. The inhibition of growth has been proven before in different systems of bacteria GHPs [11,13,21] but the mechanisms underlying this effect are still unclear. In our GO analysis, one of the most remarkable results was the inhibition of the synthesis of nucleotides by both GHPs. Nucleotides are needed for RNA and DNA synthesis, and they serve as the main energy carriers in cellular metabolism. Consequently, processes related to the transfer of information and the obtaining of energy in *Xcc* cells suffered modifications. We also highlight that both GHPs modified the synthesis and transport of components of the cell envelope. In the following sections, we will discuss the possible causes of growth arrest, the consequences of the depletion of the synthesis of ribonucleotides, and the modification of the cell envelope and the connection among these features.

## 3. Discussion

### 3.1. Transfer of Information in the Cell

FtsZ and ftsX were repressed by I3C. FtsZ is a key gene in the SOS-independent and -dependent DNA damage checkpoint [23]. FtsZ is the prokaryotic homolog of tubulin and is the first component of the cell division apparatus, which localises the division site. Once localised, ftsZ polymerises into the Z ring and provides a scaffold upon which ftsX and other components are recruited, forming the divisome. The inactivation of components of the divisome causes an arrest in cell division [24]. The SOS response is employed by bacteria upon DNA damage to ensure cell division is delayed, providing the cell with enough time for DNA repair. AITC overexpressed dnaE and I3C overexpressed dnaN, radA, and recX, which are related to DNA repair by homologous recombination (HR). Double-strand breaks can be repaired by HR, which requires the availability of an intact DNA template or by non-homologous end joining (NHEJ) if no intact template is available. Both GHPs induced ligD, which is part of NHEJ. DnaE, dnaN, radA, and recX are part of the SOS response; therefore, both treatments delayed cell division to ensure the repair of DNA by activating the SOS response. The chaperone dnaK (Hsp70) was repressed by both GHPs. The downstream chaperonin of dnaK, groL2, was repressed by I3C. Proteins that interact with dnaK involve a broad range of cellular functions including recombination and repair, translation, and ribosomal structure in *E. coli* [25]. Additionally, ftsZ is as strong dnaK binder. Therefore, bacteria retard cell division to ensure a proper repair of DNA through dnaK inhibition. The treatment of *C. jejuni* with the ITC BITC induced dnaK and groL2, which can respond to the need for protein refolding [20]. Protein refolding can be achieved through the induction of chaperone htpG (hsp90) by AITC.

RpoA was repressed and lrp was induced by I3C. Both DEGs are related to transcription. RpoA codifies the subunit α of the RNA polymerase. Lrp is a leucine-responsive regulatory protein and is a global regulator affecting the expression of many genes and operons in *E. coli* including the majority of genes expressed upon entrance into the stationary phase [26]. The translation was downregulated by I3C in the GO analysis. InfB and fusA were repressed by I3C. InfB is the translation initiation factor IF-2 and fusA is the elongation factor G [27]. Lrp and fusA can be activated under a stringent response. A stringent response is modulated by the alarmone (p)ppGpp upon starvation and in response to imbalances in the outer membrane biogenesis [28]. I3C repressed gppA, which is involved in the hydrolysis of pppGpp to ppGpp. By inhibiting gppA, the ratio pppGpp/ppGpp increases, which can provoke a higher inhibition of replication elongation [29]. AITC induced dksa. It encodes a transcription factor that binds directly to the RNA polymerase and, together with (p)ppGpp, modulates the stringent response of bacteria [30]. The conjugation of ITC with amino acids may promote a stringent response by the depletion of free amino acids in fungi and bacteria [21]. The genes responsible for the synthesis of (p)ppGpp (spoT, relA) were not significantly different from the control in any of the treatments (data not shown). With our evidence, we cannot conclude that a stringent response was activated in *Xcc* upon treatment with AITC and I3C. The growth arrest caused by both GHPs seems to be related to the need of repair DNA. Following [31], AITC is mutagenic on *E. coli* DNA, and this capability is likely related to the formation of thiobarbituric acid reactive substances and ROS. Therefore, our results suggest that the genotoxicity of both GHPs is caused in part by damage to the DNA, similar to what occurs in bacteria pathogenic to humans. Other typical responses of bacteria to ITCs, such as the activation of the stringent response, could not be confirmed with our data.

### 3.2. Energy Metabolism

Several DEGs related to glycogen and trehalose metabolism were differentially expressed by both GHPs. Both induced the debranching glycogen enzyme glgx; the branching enzymes glgz, glgb1, and glgb2; the maltosyl transferase enzymes malQ, maK, and glgE; and two DEGs related to trehalose metabolism, treA and treS. TreS transforms trehalose into maltose in a reversible reaction. Maltose can then be incorporated into α-glucans by the action of maK, malQ, glgE, and glgB [32]. TreA splits trehalose into glucose that can subsequently be taken up by the phosphotransferase-mediated uptake system. The accumulation of periplasmic trehalose increases under conditions of high osmolarity. In this case, trehalose is directed to the production of glucose and glycogen. Different in vivo and in vitro experimental evidence linked the *E. coli* stringent response with increased glycogen content and an enhanced expression of glg genes at the onset of the stationary phase [33].

**Figure 7:**
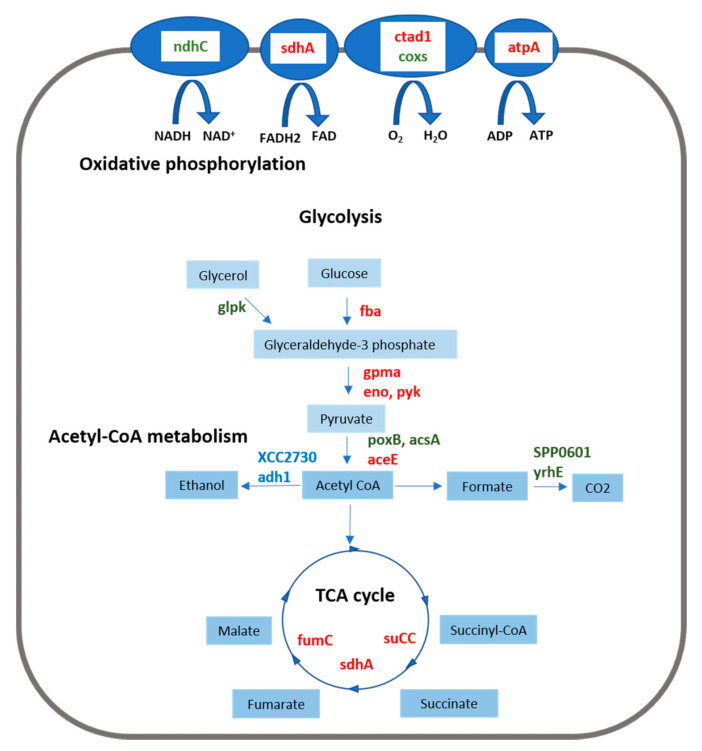
Schematic diagram representing DEGs integrated in the KEGG categories of glycolysis-gluconeogenesis, acetyl-CoA metabolism, citrate cycle, and energy-oxidative phosphorylation. DEGs highlighted in red are downregulated by I3C, whereas those highlighted in blue and green are upregulated by I3C and AITC, respectively. A description of DEGs is provided in Supplementary Tables S1 and S2.

In the general GO analysis, both treatments inhibited the aerobic respiration. In the comparison of AITC vs. I3C, this inhibition was stronger with I3C than with AITC. I3C inhibits DEGs related to glycolysis, the TCA cycle, and oxidative phosphorylation (Figure 7). AITC induces the conversion of glycerol to glyceraldehyde-3-phosphate, the production of acetyl-CoA from pyruvate, and its use in formate synthesis and decarboxylation (Figure 7) with the obtaining of NADH. Formate is produced in significant amounts under anaerobic and microaerobic conditions in *E. coli* and may perform a significant role in the antioxidative stress defence in the stationary phase [34]. The regeneration of NAD^+^ in both cases is produced through fermentation by the induction of the alcohol dehydrogenases adh1 and XCC2730 by I3C and AITC, respectively.

ITCs damage mitochondria membranes and consequently inhibit oxygen-dependent respiration and ATPase activity in eukaryotic cells [35]. Similarly, in *E. coli*, treatment with 2-(4-hydro-xyphenyl) ethyl ITC decreased the cellular content of ATP. In the microaerophile *C. jejuni*, the inhibition of O_2_ respiration by ITCs provokes an upregulation of fumarate and nitrate respiration [20]. Our results suggest that both GHPs inhibit the growth of *Xcc* and provoke the entrance into a state of low energy, inducing several genes (lrp, glg genes) that are normally activated when bacteria enter the stationary phase. The inhibition of O_2_ respiration may be a response to damage in the cell membrane, similar to what occurs in the mitochondrial membrane in eukaryotic cells.

### 3.3. Cell Envelope

Several DEGs induced by both GHPs were related to the cell envelope. The envelope of Gram-negative bacteria contains two membranes: the inner membrane surrounds the cytoplasmic components and the outer membrane separates the cell from its environment. These two membranes surround the periplasm, which contains the peptidoglycan cell wall. The outer membrane contains phospholipids in the inner leaflet and lipopolysaccharides in the outer leaflet [36]. The Lpt complex mediates the transport of lipopolysaccharides and its insertion and assembly at the outer membrane [37]. LptD, which is part of the Lpt complex, was induced by AITC.

Outer membrane proteins and lipoproteins are synthesised as pre-proteins in the cytoplasm and then secreted across the inner membrane by the SEC translocase that transports unfolded proteins. SecD and secF, parts of the Sec complex, were induced by both GHPs. LolC and bamb were induced by AITC. LolC is an ABC transporter that mediates the transfer of lipoproteins to the outer membrane [38]. The Bam complex is responsible for the assembly of β-barrel proteins into the outer membrane.

MrcA, nlpD, and tolB were induced by I3C. MrcA codifies for a membrane D-D carboxypeptidase, which possesses peptidoglycan glycosyltransferase activity and affects the degree of cross-linking of cell wall peptidoglycans [39]. NlpD is a membrane protein and a key regulator of the initiation of the stationary phase and stress response in *E. coli* [39]. TolB is a periplasmic protein interacting with Pal, the peptidoglycan-associated lipoprotein anchored to the outer membrane. Mutants of tolB promoted both envelope stress and deformation of the membrane. This causes the inhibition of the electron transport chain, energy production, and the formation of the membrane potential necessary for nutrient import in *E. coli* [40].

AITC induces the transport of lipopolysaccharides to the outer membrane. I3C induces the cross-linking of peptidoglycan and both induce the transport of lipoproteins to the outer membrane. All these movements can respond to the need of repairing the cell envelope. Accordingly, AITC provokes damage on the cell membrane in *E. coli* in addition to affecting the hydrophobic character of lipopolysaccharides of their outer membrane [21,22]. Agreeing with this hypothesis, I3C induced accC and fabA, which participate in the Type II fatty acid synthesis of bacteria. AccC is part of the acetyl-CoA carboxylase complex, which initiates the synthesis. FabA is responsible together with fabB for the synthesis of unsaturated fatty acids in *E. coli* [41]. *E. coli* and *S. aureus* treated with the antibiotic naringenin induced fabA to increase unsaturated fatty acids to increase membrane fluidity [42]. Therefore, the modification of the synthesis of lipids can be produced to increase the fluidity of the cellular membrane.

Synthesis and the transport of components of the cell envelope were induced by both GHPs, suggesting that components of the cell envelope were damaged, in agreement with the genotoxic mechanisms of ITCs in eukaryotic cells [43,44]. Damage in cell membranes can cause the inhibition of O_2_ respiration.

### 3.4. Detoxification and Biofilm Formation

The conjugation of ITCs with glutathione represents a principal metabolic route of detoxification [21]. GST enzymes catalyse the conjugation of GSH to the electrophilic centres of ITCs. The resulting conjugates are more water soluble and can be further metabolised and excreted. Similarly, a GST-encoding gene was identified as being upregulated after the treatment of *P. aeruginosa* with the ITC iberin [45]. Agreeing with these results, gst1 was induced in *Xcc* treated with AITC. ITCs can disrupt redox homeostasis and increase the content of ROS in cells [11]; consequently, antioxidant defences increase upon treatment with ITCs. *Xcc* treated with AITC induced several antioxidant enzymes: oxyR, katE, sodC1, sodC2, ahpC. KatE, ahpC, and oxyR are involved in the detoxification of hydrogen peroxides, whereas sodC1 and sodC2 are involved in the detoxification of superoxides. KatE and sodC2 were also induced by the indole I3C.

*Xcc* treated with GHPs induced multidrug efflux system components in a specific manner. MexE and mdtB were induced by AITC, whereas macB was induced by I3C. Efflux systems may promote the extrusion of GHPs from the cell [21]. Therefore, *Xcc*-induced detoxification mechanisms such as the antioxidant defence system and the multidrug efflux system to cope with the toxic effects driven by both chemicals.

Several ITCs including AITC inhibit the biofilm formation of the human pathogens *P. aeruginosa*, *S. aureus*, and *L. monocytogenes* [17]. The inhibition of biofilms is presumably due to interferences in bacterial viability, motility, and surface properties. I3C induced pdeA, which catalyses the hydrolysis of cyclic-di-GMP (c-di-GMP) to 5′-pGpG. c-di-GMP is a global bacterial second messenger that modulates surface adaptation, biofilm formation, cell cycle progression, and virulence [46]. I3C inhibited cheB2. The inhibition of this chemotaxis gene causes modifications in the mobility of *E. coli* [47]. Therefore, by inducing the hydrolysis of c-di-GMP and inhibiting cheB2, I3C probably modifies biofilm formation, which may have consequences for the pathogenicity of the bacteria.

## 4. Materials and Methods

### 4.1. Bacteria Culture and GHP Treatment

*Xcc* race 1 strain HRI3811 was provided by Warwick HRI (Wellesbourne, U.K.). Bacteria colonies were plated on Petri dishes containing potato dextrose agar (PDA) (Sigma-Aldrich, St. Louis, MO, USA) and incubated at 30 °C for 24 h. A loop of bacterial growth was then subcultured in Luria Bertani medium (LB) (Sigma-Aldrich, St. Louis, MO, USA) overnight in a shaker at 30 °C and in the dark. This was employed as a pre-inoculum to monitor the growth of *Xcc*. The following day, 1 mL of pre-inoculum was supplemented with 2 mL of fresh media, then 10 μL of GHPs dissolved in DMSO was added to the medium to reach a final concentration of 0, 25, 50, 75, 100, and 125 μM. Allyl-ITC (AITC) (36682) and indol-3-carbinol (I3C) (I7256-5G) were obtained from Sigma-Aldrich (St. Louis, MO, USA). The turbidity of the control and the treated samples was monitored over six hours, taking measurements every second hour using a microplate spectrophotometer (Spectra MR; Dynex Technologies, Chantilly, VA, USA) at 600 nm. Bacteria were cultivated in 12-well plates at 30 °C with continuous shaking during the whole experiment. The control bacteria were monitored by employing identical conditions. Three repetitions were carried out for each treatment. To characterize the phenotype of growth, we took an aliquot of the samples treated with 100 μM of each treatment and made 1/10 dilutions that were cultured in LBA medium.

### 4.2. RNA Isolation, Library Preparation, and Sequencing

Bacteria supplemented with both GHPs (100 μM) and controls were grown as described in the previous section. After four hours of log-phase growing, the bacteria were precipitated by centrifugation to perform RNA extractions. Each treatment was repeated twice. RNA was extracted with an RNAspin Mini RNA isolation kit (GE Healthcare, Little Chalfont, UK) according to the manufacturer’s protocol. RNA quantity and quality were assessed using a NanoDrop Spectrophotometer (NDS) before proceeding with RNA-seq. To remove any traces of genomic DNA, the RNA was treated with DNase following the manufacturer’s instructions. Two repetitions were performed for each treatment.

RNA sequencing was performed with GenXPro’s pipeline (GenXPro GmbH, Frankfurt, Germany). Briefly, the ribosomal RNA was depleted using RiboMinus Bacteria Probe Mix v2 (Thermo Fisher). The remaining RNA was used to generate RNA-seq libraries using the NEBNextUltra RNA Library Prep Kit for Illumina, according to the manual. The samples were sequenced using an Illumina HiSeq 2000 version 4 chemistry (Illumina, Inc., San Diego, CA, USA). The reads were trimmed for low quality and adapter residues were cut off. The reads were de novo assembled using Cap3, and the contigs were then BLASTed vs. the bacterial database (ENSEMBL). The transcripts were quantified after mapping the reads to the annotated contigs. The statistical analysis was performed using the DEseq R package according to [48].

### 4.3. Functional Analysis

To provide a general overview of the results, a GO analysis with all the annotated transcripts was conducted for each comparison (GHPs vs. the control and AITC vs. I3C) with GO-Tool2. The likelihood of a specific GO term was calculated using Fisher’s exact test. The analysis was based on the R-package TopGO. Only those GO terms with a *p*-value < 0.05 and −1 < ratio > 1 were considered in the discussion.

Transcripts with a false discovery rate (FDR) < 0.05 and −1 < log_2_ fold change (FC) > 1 were considered to be differentially expressed genes (DEGs) between treatments. DEGs were localised into KEGG pathways (https://www.genome.jp/kegg/pathway.html, 29 January 2021) to discover interconnections among them. Annotations and relationships among DEGs were deduced employing the genome of *Xcc* ATCC 33913 as the reference.

### 4.4. Quantitative Reverse-Transcription-PCR (RT-qPCR) Validation

*Xcc* was cultured as described above with the corresponding GHPs at a concentration of 100 µM. The procedure for RNA extraction was the same as that followed in the RNA sequencing analysis. Three replicates were performed for each treatment. All primer pairs used are listed in Table S3. One microgram of total RNA was reverse-transcribed using the GoScript™ reverse-transcription system and random oligos (Promega, Madison, WI, USA). RT-qPCR was performed in a 20 μL reaction with the Fast Start Universe SYBR Green Master (ROX) mix (Roche Molecular Systems Inc., Pleasanton, CA, USA), following the manufacturer’s instructions. The DNA gyrase subunit B transcript was used as housekeeping gene [49]. RT-qPCRs were carried out on a 7500 Real-Time PCR System (Applied Biosystem, Forster City, CA, USA), and the primer efficiency was calculated using LingRegPCR software [50]. Efficiencies were used to calculate the relative gene expression using the ΔΔCt method [51]. The statistical significance was calculated using Fisher’s least significant difference (LSD) at the 0.05 level of probability to compare the relative gene expression in the GHP-treated samples vs. the control.

## 5. Conclusions

We conducted a study on the transcriptomic reprogramming of *Xcc* after being supplemented with one aliphatic ITC and one indole. Both treatments retarded the growth of the bacteria compared to the control. At the transcriptomic level, the cell division was stopped by activating the SOS response through dnaK chaperone inhibition. The transcriptomic reprograming of the cell envelope was carried out by *Xcc* treated with both GHPs by inducing the transport of lipopolysaccharides and lipoproteins to the outer membrane and increasing the cross-linking of peptidoglycan. An arrest in *Xcc* growth seems to be related to the need to stop cell division to repair damaged DNA and cell envelope components. GHPs modified energy metabolism by inhibiting the aerobic respiration. As *Xcc* growth is slower, the need for energy is lower and cell resources are mobilised to synthesise glycogen. *Xcc*-induced detoxification mechanisms such as the antioxidant defence system and the multidrug efflux system to cope with the toxic effects driven by GHPs. This is the first time that the transcriptomic response of a plant pathogenic bacteria to treatment with GHPs derived from the secondary metabolites, GSLs, has been studied. This information will allow us to better understand the interaction of plant pathogens mediated by GSLs.

## Data Availability

The datasets analysed for this study can be found in the Gene Expression Omnibus repository with the accession number GSE167555.

## Supplementary Materials

The following are available online at https://www.mdpi.com/article/10.3390/plants10081656/s1, Table S1: Upregulated differentially expressed genes in the comparisons AITC vs. Control and I3C vs. Control. Table S2: Downregulated differentially expressed genes in the comparisons AITC vs. Control and I3C vs. Control. Table S3: Primers used for RT-qPCR.
