# Supplementary material for: Transcriptomic Reprograming of Xanthomonas campestris pv. campestris after Treatment with Hydrolytic Products Derived from Glucosinolates
Source: Plants (Basel). 2021 Aug 11;10(8):1656. doi: 10.3390/plants10081656 (PMC8400333; doi:10.3390/plants10081656)
Supplement: Supplementary file 1 [file plants-10-01656-s001.zip › supplementary/Table S1.pdf]

List of up-regulated DEGs in the comparison AITCvsControl			
Gene	Description	fdr	log2fc
speA	sp|Q8P448|SPEA_XANCP Biosynthetic arginine decarboxylase OS=Xanthomonas campestris pv. campestris (strain ATCC 33913 / NCPPB 528 / LMG 568) GN=speA PE=3 SV=2	0.0307	1.7199
sodC1	sp|O67149|SODC1_AQUAE Superoxide dismutase [Cu-Zn] 1 OS=Aquifex aeolicus (strain VF5) GN=sodC1 PE=3 SV=1	0.0393	1.7619
rne	tr|Q5GY94|Q5GY94_XANOR Ribonuclease E OS=Xanthomonas oryzae pv. oryzae (strain KACC10331 / KXO85) GN=rne PE=3 SV=1	0.0480	1.7895
rplB	sp|Q9PE73|RL2_XYLFA 50S ribosomal protein L2 OS=Xylella fastidiosa (strain 9a5c) GN=rplB PE=3 SV=1	0.0294	1.8293
fadJ	sp|Q5QXM1|FADJ_IDILO Fatty acid oxidation complex subunit alpha OS=Idiomarina loihiensis (strain ATCC BAA-735 / DSM 15497 / L2-TR) GN=fadJ PE=3 SV=1	0.0434	1.8468
ispH	sp|Q3BW40|ISPH_XANC5 4-hydroxy-3-methylbut-2-enyl diphosphate reductase OS=Xanthomonas campestris pv. vesicatoria (strain 85-10) GN=ispH PE=3 SV=1	0.0298	1.8895
glgX	sp|Q3YW94|GLGX_SHISS Glycogen debranching enzyme OS=Shigella sonnei (strain Ss046) GN=glgX PE=3 SV=1	0.0407	1.9060
SERP0500	sp|Q5HQP8|Y500_STAEQ UPF0051 protein SERP0500 OS=Staphylococcus epidermidis (strain ATCC 35984 / RP62A) GN=SERP0500 PE=3 SV=1	0.0229	1.9394
sodC2	sp|O66602|SODC2_AQUAE Superoxide dismutase [Cu-Zn] 2 OS=Aquifex aeolicus (strain VF5) GN=sodC2 PE=3 SV=1	0.0436	1.9460
ychF	sp|Q7VMI2|YCHF_HAEDU Ribosome-binding ATPase YchF OS=Haemophilus ducreyi (strain 35000HP / ATCC 700724) GN=ychF PE=3 SV=3	0.0449	1.9735
Alvin_0064	sp|P45373|Y064_ALLVD Uncharacterized protein Alvin_0064 OS=Allochromatium vinosum (strain ATCC 17899 / DSM 180 / NBRC 103801 / NCIMB 10441 / D) GN=Alvin_0064 PE=4 SV=1	0.0449	1.9768
dxr	sp|B0RW72|DXR_XANCB 1-deoxy-D-xylulose 5-phosphate reductoisomerase OS=Xanthomonas campestris pv. campestris (strain B100) GN=dxr PE=3 SV=1	0.0094	1.9779
XCC0317	tr|Q8PDL8|Q8PDL8_XANCP Uncharacterized protein OS=Xanthomonas campestris pv. campestris (strain ATCC 33913 / NCPPB 528 / LMG 568) GN=XCC0317 PE=4 SV=1	0.0216	1.9941
XCC1489	tr|Q8PAJ4|Q8PAJ4_XANCP Uncharacterized protein OS=Xanthomonas campestris pv. campestris (strain ATCC 33913 / NCPPB 528 / LMG 568) GN=XCC1489 PE=4 SV=1	0.0273	2.0018
guaA	sp|Q8P8Q6|GUAA_XANCP GMP synthase [glutamine-hydrolyzing] OS=Xanthomonas campestris pv. campestris (strain ATCC 33913 / NCPPB 528 / LMG 568) GN=guaA PE=3 SV=1	0.0449	2.0161
yrhE	sp|Q795Y4|FDHL_BACSU Putative formate dehydrogenase YrhE OS=Bacillus subtilis (strain 168) GN=yrhE PE=3 SV=1	0.0185	2.0240
PSPPH_2483	sp|Q48IV1|LOVHK_PSE14 Blue-light-activated protein OS=Pseudomonas syringae pv. phaseolicola (strain 1448A / Race 6) GN=PSPPH_2483 PE=3 SV=2	0.0194	2.0372
dnaA	sp|Q8PEH5|DNAA_XANCP Chromosomal replication initiator protein DnaA OS=Xanthomonas campestris pv. campestris (strain ATCC 33913 / NCPPB 528 / LMG 568) GN=dnaA PE=3 SV=1	0.0449	2.0374

malQ	tr|B0RMT9|B0RMT9_XANCB MalQ protein OS=Xanthomonas campestris pv. campestris (strain B100) GN=malQ PE=4 SV=1	0.0441	2.0541
araJ	tr|Q8PB10|Q8PB10_XANCP MFS transporter OS=Xanthomonas campestris pv. campestris (strain ATCC 33913 / NCPPB 528 / LMG 568) GN=araJ PE=4 SV=1	0.0369	2.0608
yagR	tr|Q8P786|Q8P786_XANCP Oxidoreductase OS=Xanthomonas campestris pv. campestris (strain ATCC 33913 / NCPPB 528 / LMG 568) GN=yagR PE=4 SV=1	0.0297	2.0653
phaF	tr|Q8PAA2|Q8PAA2_XANCP Poly(Hydroxyalcanoate) granule associated protein OS=Xanthomonas campestris pv. campestris (strain ATCC 33913 / NCPPB 528 / LMG 568) GN=phaF PE=4 SV=1	0.0410	2.0683
ligD	sp|A0R3R7|LIGD_MYCS2 Multifunctional non-homologous end joining protein LigD OS=Mycobacterium smegmatis (strain ATCC 700084 / mc(2)155) GN=ligD PE=1 SV=2	0.0401	2.0777
corA	sp|Q9WZ31|CORA_THEMA Magnesium transport protein CorA OS=Thermotoga maritima (strain ATCC 43589 / MSB8 / DSM 3109 / JCM 10099) GN=corA PE=1 SV=1	0.0449	2.1074
htpG	sp|Q4UVY7|HTPG_XANC8 Chaperone protein HtpG OS=Xanthomonas campestris pv. campestris (strain 8004) GN=htpG PE=3 SV=1	0.0205	2.1085
betA	sp|Q8P5D7|BETA_XANCP Oxygen-dependent choline dehydrogenase OS=Xanthomonas campestris pv. campestris (strain ATCC 33913 / NCPPB 528 / LMG 568) GN=betA PE=3 SV=1	0.0265	2.1108
metN1	sp|Q02ME3|METN1_PSEAB Methionine import ATP-binding protein MetN 1 OS=Pseudomonas aeruginosa (strain UCBPP-PA14) GN=metN1 PE=3 SV=1	0.0357	2.1159
mip	sp|Q5ZXE0|MIP_LEGPH Outer membrane protein MIP OS=Legionella pneumophila subsp. pneumophila (strain Philadelphia 1 / ATCC 33152 / DSM 7513) GN=mip PE=1 SV=2	0.0169	2.1213
ahcY	sp|Q8PCH5|SAHH_XANCP Adenosylhomocysteinase OS=Xanthomonas campestris pv. campestris (strain ATCC 33913 / NCPPB 528 / LMG 568) GN=ahcY PE=3 SV=1	0.0190	2.1314
secD	sp|O33517|SECD_RHOCB Protein translocase subunit SecD OS=Rhodobacter capsulatus (strain ATCC BAA-309 / NBRC 16581 / SB1003) GN=secD PE=3 SV=1	0.0317	2.1335
HI_1208	sp|P71373|Y1208_HAEIN Epimerase family protein HI_1208 OS=Haemophilus influenzae (strain ATCC 51907 / DSM 11121 / KW20 / Rd) GN=HI_1208 PE=3 SV=1	0.0338	2.1373
rpmJ2	sp|Q1H1T1|RL362_METFK 50S ribosomal protein L36 2 OS=Methylobacillus flagellatus (strain KT / ATCC 51484 / DSM 6875) GN=rpmJ2 PE=3 SV=1	0.0509	2.1600
dksA	sp|B8H0C0|DKSA_CAUCN RNA polymerase-binding transcription factor DksA OS=Caulobacter crescentus (strain NA1000 / CB15N) GN=dksA PE=3 SV=1	0.0334	2.1632
acsA	sp|Q8P3L1|ACSA_XANCP Acetyl-coenzyme A synthetase OS=Xanthomonas campestris pv. campestris (strain ATCC 33913 / NCPPB 528 / LMG 568) GN=acsA PE=3 SV=1	0.0449	2.1678
pheS	sp|Q8P7Z5|SYFA_XANCP Phenylalanine--tRNA ligase alpha subunit OS=Xanthomonas campestris pv. campestris (strain ATCC 33913 / NCPPB 528 / LMG 568) GN=pheS PE=3 SV=1	0.0297	2.1768

yagR	tr|Q8P786|Q8P786_XANCP Oxidoreductase OS=Xanthomonas campestris pv. campestris (strain ATCC 33913 / NCPPB 528 / LMG 568) GN=yagR PE=4 SV=1	0.0329	2.1856
stcV	sp|Q00727|STCV_EMENI Putative sterigmatocystin biosynthesis dehydrogenase stcV OS=Emericella nidulans (strain FGSC A4 / ATCC 38163 / CBS 112.46 / NRRL 194 / M139) GN=stcV PE=3 SV=2	0.0307	2.1882
VC0395_0473	sp|A5F0B6|Y473_VIBC3 UPF0312 protein VC0395_0473/VC395_A0785 OS=Vibrio cholerae serotype O1 (strain ATCC 39541 / Classical Ogawa 395 / O395) GN=VC0395_0473 PE=3 SV=1	0.0281	2.2040
queG	sp|Q8P8E0|QUEG_XANCP Epoxyqueuosine reductase OS=Xanthomonas campestris pv. campestris (strain ATCC 33913 / NCPPB 528 / LMG 568) GN=queG PE=3 SV=1	0.0469	2.2223
XCC2823	tr|Q8P6Z2|Q8P6Z2_XANCP Uncharacterized protein OS=Xanthomonas campestris pv. campestris (strain ATCC 33913 / NCPPB 528 / LMG 568) GN=XCC2823 PE=4 SV=1	0.0269	2.2249
tsaC	sp|Q8P4F2|TSAC_XANCP Threonylcarbamoyl-AMP synthase OS=Xanthomonas campestris pv. campestris (strain ATCC 33913 / NCPPB 528 / LMG 568) GN=tsaC PE=3 SV=1	0.0378	2.2276
NMB0075	sp|Q51152|YHGF_NEIMB Uncharacterized protein NMB0075 OS=Neisseria meningitidis serogroup B (strain MC58) GN=NMB0075 PE=3 SV=2	0.0230	2.2359
XCC0864	tr|Q8PC71|Q8PC71_XANCP Transcriptional regulator OS=Xanthomonas campestris pv. campestris (strain ATCC 33913 / NCPPB 528 / LMG 568) GN=XCC0864 PE=4 SV=1	0.0449	2.2362
argG	sp|Q8P8J4|ASSY_XANCP Argininosuccinate synthase OS=Xanthomonas campestris pv. campestris (strain ATCC 33913 / NCPPB 528 / LMG 568) GN=argG PE=3 SV=1	0.0307	2.2464
RP373	sp|Q9ZDF6|MAO2_RICPR Probable NADP-dependent malic enzyme OS=Rickettsia prowazekii (strain Madrid E) GN=RP373 PE=3 SV=1	0.0257	2.2481
gcvP	sp|Q8PBK7|GCSP_XANCP Glycine dehydrogenase (decarboxylating) OS=Xanthomonas campestris pv. campestris (strain ATCC 33913 / NCPPB 528 / LMG 568) GN=gcvP PE=3 SV=1	0.0259	2.2551
XCR_3563	tr|G0CEF3|G0CEF3_XANCA Tetratricopeptide repeat domain protein OS=Xanthomonas campestris pv. raphani 756C GN=XCR_3563 PE=4 SV=1	0.0279	2.2628
acsA	sp|Q8P3L1|ACSA_XANCP Acetyl-coenzyme A synthetase OS=Xanthomonas campestris pv. campestris (strain ATCC 33913 / NCPPB 528 / LMG 568) GN=acsA PE=3 SV=1	0.0173	2.2650
XCC2951	tr|Q8P6L6|Q8P6L6_XANCP Pseudouridylate synthase OS=Xanthomonas campestris pv. campestris (strain ATCC 33913 / NCPPB 528 / LMG 568) GN=XCC2951 PE=4 SV=1	0.0183	2.2800
XCC3527	tr|Q8P521|Q8P521_XANCP Endonuclease OS=Xanthomonas campestris pv. campestris (strain ATCC 33913 / NCPPB 528 / LMG 568) GN=XCC3527 PE=4 SV=1	0.0354	2.2994
acsA	sp|Q8P3L1|ACSA_XANCP Acetyl-coenzyme A synthetase OS=Xanthomonas campestris pv. campestris (strain ATCC 33913 / NCPPB 528 / LMG 568) GN=acsA PE=3 SV=1	0.0210	2.3021
mexE	tr|Q8PDB9|Q8PDB9_XANCP Component of multidrug efflux system OS=Xanthomonas campestris pv. campestris (strain ATCC 33913 / NCPPB 528 / LMG 568) GN=mexE PE=4 SV=1	0.0025	2.3098

wxcM	tr|B0RVL6|B0RVL6_XANCB WxcM protein OS=Xanthomonas campestris pv. campestris (strain B100) GN=wxcM PE=4 SV=1	0.0466	2.3208
treA	sp|Q8P519|TREA_XANCP Periplasmic trehalase OS=Xanthomonas campestris pv. campestris (strain ATCC 33913 / NCPPB 528 / LMG 568) GN=treA PE=3 SV=1	0.0396	2.3275
XCC3994	tr|Q8P3S5|Q8P3S5_XANCP Uncharacterized protein OS=Xanthomonas campestris pv. campestris (strain ATCC 33913 / NCPPB 528 / LMG 568) GN=XCC3994 PE=4 SV=1	0.0331	2.3331
secF	tr|G7TBP2|G7TBP2_XANOB Protein-export membrane protein SecF OS=Xanthomonas oryzae pv. oryzicola (strain BLS256) GN=secF PE=3 SV=1	0.0177	2.3346
rlmN	sp|B0RT51|RLMN_XANCB Dual-specificity RNA methyltransferase RlmN OS=Xanthomonas campestris pv. campestris (strain B100) GN=rlmN PE=3 SV=1	0.0307	2.3420
mak	sp|A5TYK2|MAK_MYCTA Maltokinase OS=Mycobacterium tuberculosis (strain ATCC 25177 / H37Ra) GN=mak PE=3 SV=1	0.0024	2.3448
XCC1433	tr|Q8PAP6|Q8PAP6_XANCP Uncharacterized protein OS=Xanthomonas campestris pv. campestris (strain ATCC 33913 / NCPPB 528 / LMG 568) GN=XCC1433 PE=4 SV=1	0.0056	2.3494
ahcY	sp|Q8PCH5|SAHH_XANCP Adenosylhomocysteinase OS=Xanthomonas campestris pv. campestris (strain ATCC 33913 / NCPPB 528 / LMG 568) GN=ahcY PE=3 SV=1	0.0373	2.3541
PROSTU_00109	tr|B2PU91|B2PU91_PROST Uncharacterized protein OS=Providencia stuartii ATCC 25827 GN=PROSTU_00109 PE=4 SV=1	0.0216	2.3659
uvrC	sp|Q8P8W9|UVRC_XANCP UvrABC system protein C OS=Xanthomonas campestris pv. campestris (strain ATCC 33913 / NCPPB 528 / LMG 568) GN=uvrC PE=3 SV=1	0.0412	2.3694
pqqB	sp|Q8P6N0|PQQB_XANCP Coenzyme PQQ synthesis protein B OS=Xanthomonas campestris pv. campestris (strain ATCC 33913 / NCPPB 528 / LMG 568) GN=pqqB PE=3 SV=1	0.0396	2.3722
tsf	sp|Q87A70|EFTS_XYLFT Elongation factor Ts OS=Xylella fastidiosa (strain Temecula1 / ATCC 700964) GN=tsf PE=3 SV=1	0.0387	2.3738
XCC0267	tr|Q8PDR8|Q8PDR8_XANCP Uncharacterized protein OS=Xanthomonas campestris pv. campestris (strain ATCC 33913 / NCPPB 528 / LMG 568) GN=XCC0267 PE=4 SV=1	0.0219	2.3853
oma1	sp|Q9P7G4|OMA1_SCHPO Mitochondrial metalloendopeptidase OMA1 OS=Schizosaccharomyces pombe (strain 972 / ATCC 24843) GN=oma1 PE=3 SV=1	0.0341	2.3876
coxS	sp|P19921|DCMS_OLICO Carbon monoxide dehydrogenase small chain OS=Oligotropha carboxidovorans (strain ATCC 49405 / DSM 1227 / OM5) GN=coxS PE=1 SV=2	0.0120	2.3912
csd	sp|Q9PDA6|CSD_XYLFA Probable cysteine desulfurase OS=Xylella fastidiosa (strain 9a5c) GN=csd PE=3 SV=1	0.0387	2.3936
ndhC	sp|Q2JT70|NU3C_SYNJA NAD(P)H-quinone oxidoreductase subunit 3 OS=Synechococcus sp. (strain JA-3-3Ab) GN=ndhC PE=3 SV=1	0.0479	2.3984
SPCC757.02c	sp|O74913|YJ72_SCHPO Uncharacterized protein C757.02c OS=Schizosaccharomyces pombe (strain 972 / ATCC 24843) GN=SPCC757.02c PE=4 SV=1	0.0345	2.4043

glnA	tr|Q8P897|Q8P897_XANCP Glutamine synthetase OS=Xanthomonas campestris pv. campestris (strain ATCC 33913 / NCPPB 528 / LMG 568) GN=glnA PE=3 SV=1	0.0230	2.4098
XCC4195	tr|Q8P383|Q8P383_XANCP Uncharacterized protein OS=Xanthomonas campestris pv. campestris (strain ATCC 33913 / NCPPB 528 / LMG 568) GN=XCC4195 PE=4 SV=1	0.0191	2.4117
poxB	tr|Q8PDX8|Q8PDX8_XANCP Pyruvate dehydrogenase OS=Xanthomonas campestris pv. campestris (strain ATCC 33913 / NCPPB 528 / LMG 568) GN=poxB PE=3 SV=1	0.0298	2.4119
dxs	sp|Q8P815|DXS_XANCP 1-deoxy-D-xylulose-5-phosphate synthase OS=Xanthomonas campestris pv. campestris (strain ATCC 33913 / NCPPB 528 / LMG 568) GN=dxs PE=3 SV=1	0.0292	2.4127
mltA	tr|V7Z8N0|V7Z8N0_9XANT Transglycosylase associated protein OS=Xanthomonas hortorum pv. carotae str. M081 GN=mltA PE=4 SV=1	0.0307	2.4133
ligD	sp|A0R3R7|LIGD_MYCS2 Multifunctional non-homologous end joining protein LigD OS=Mycobacterium smegmatis (strain ATCC 700084 / mc(2)155) GN=ligD PE=1 SV=2	0.0033	2.4153
MIMI_R526	sp|Q5UQ83|YR526_MIMIV Putative alpha/beta hydrolase R526 OS=Acanthamoeba polyphaga mimivirus GN=MIMI_R526 PE=1 SV=1	0.0259	2.4326
SPCC13B11.04c	sp|O74540|FADH2_SCHPO Putative S-(hydroxymethyl)glutathione dehydrogenase 2 OS=Schizosaccharomyces pombe (strain 972 / ATCC 24843) GN=SPCC13B11.04c PE=3 SV=2	0.0183	2.4329
XCC0007	tr|Q8PEG9|Q8PEG9_XANCP Uncharacterized protein OS=Xanthomonas campestris pv. campestris (strain ATCC 33913 / NCPPB 528 / LMG 568) GN=XCC0007 PE=4 SV=1	0.0088	2.4363
sotB	sp|B1J9Y3|SOTB_PSEPW Probable sugar efflux transporter OS=Pseudomonas putida (strain W619) GN=sotB PE=3 SV=1	0.0239	2.4411
Nmul_A2370	sp|Q2Y6G2|GPH_NITMU Phosphoglycolate phosphatase OS=Nitrosospira multiformis (strain ATCC 25196 / NCIMB 11849) GN=Nmul_A2370 PE=3 SV=1	0.0219	2.4429
hemY	tr|Q8PDY8|Q8PDY8_XANCP Porphyrin biosynthesis protein OS=Xanthomonas campestris pv. campestris (strain ATCC 33913 / NCPPB 528 / LMG 568) GN=hemY PE=4 SV=1	0.0222	2.4429
oprF	sp|P37726|PORF_PSEFL Outer membrane porin F OS=Pseudomonas fluorescens GN=oprF PE=1 SV=1	0.0127	2.4446
ihfB	sp|Q9PAQ8|IHFB_XYLFA Integration host factor subunit beta OS=Xylella fastidiosa (strain 9a5c) GN=ihfB PE=3 SV=2	0.0276	2.4493
oxyR	sp|P52678|OXYR_MYCLE Probable hydrogen peroxide-inducible genes activator OS=Mycobacterium leprae (strain TN) GN=oxyR PE=3 SV=1	0.0329	2.4526
araB	sp|Q1JUP5|ARALA_AZOBR L-arabinolactonase OS=Azospirillum brasilense GN=araB PE=1 SV=1	0.0087	2.4647
csd	sp|Q9PDA6|CSD_XYLFA Probable cysteine desulfurase OS=Xylella fastidiosa (strain 9a5c) GN=csd PE=3 SV=1	0.0414	2.4824
SPCC757.02c	sp|O74913|YJ72_SCHPO Uncharacterized protein C757.02c OS=Schizosaccharomyces pombe (strain 972 / ATCC 24843) GN=SPCC757.02c PE=4 SV=1	0.0177	2.4946
phS	tr|Q8P523|Q8P523_XANCP Histidine kinase/response regulator hybrid protein OS=Xanthomonas campestris pv. campestris (strain ATCC 33913 / NCPPB 528 / LMG 568) GN=phS PE=4 SV=1	0.0173	2.4983
dnaB	sp|Q8K932|DNAB_BUCAP Replicative DNA helicase OS=Buchnera aphidicola subsp. Schizaphis graminum (strain Sg) GN=dnaB PE=3 SV=1	0.0021	2.5109

XCC4164	tr|Q8P3B2|Q8P3B2_XANCP Uncharacterized protein OS=Xanthomonas campestris pv. campestris (strain ATCC 33913 / NCPPB 528 / LMG 568) GN=XCC4164 PE=4 SV=1	0.0009	2.5389
TOP1E	sp|Q7T6X9|TOP1E_MIMIV DNA topoisomerase 1B OS=Acanthamoeba polyphaga mimivirus GN=TOP1E PE=1 SV=2	0.0278	2.5443
XCAW_00637	tr|M4WC88|M4WC88_XANCI UPF0391 membrane protein XCAW_00637 OS=Xanthomonas citri subsp. citri Aw12879 GN=XCAW_00637 PE=3 SV=1	0.0342	2.5463
ttgH	sp|Q93PU4|TTGH_PSEPT Toluene efflux pump membrane transporter TtgH OS=Pseudomonas putida (strain DOT-T1E) GN=ttgH PE=2 SV=2	0.0219	2.5478
dhfrIII	sp|P12833|DYR3_SALTM Dihydrofolate reductase type 3 OS=Salmonella typhimurium GN=dhfrIII PE=1 SV=1	0.0183	2.5615
prfC	sp|B8GTY2|RF3_THISH Peptide chain release factor 3 OS=Thioalkalivibrio sulfidiphilus (strain HL-EbGR7) GN=prfC PE=3 SV=1	0.0373	2.5617
SSP0601	sp|Q49ZN0|FDHL_STAS1 Putative formate dehydrogenase SSP0601 OS=Staphylococcus saprophyticus subsp. saprophyticus (strain ATCC 15305 / DSM 20229) GN=SSP0601 PE=3 SV=1	0.0066	2.5620
lolC	sp|Q8K9N8|LOLC_BUCAP Lipoprotein-releasing system transmembrane protein LolC OS=Buchnera aphidicola subsp. Schizaphis graminum (strain Sg) GN=lolC PE=3 SV=1	0.0140	2.5620
rplU	sp|Q9PAS1|RL21_XYLFA 50S ribosomal protein L21 OS=Xylella fastidiosa (strain 9a5c) GN=rplU PE=3 SV=1	0.0361	2.5684
ubiB	sp|B0RLZ0|UBIB_XANCB Probable protein kinase UbiB OS=Xanthomonas campestris pv. campestris (strain B100) GN=ubiB PE=3 SV=1	0.0026	2.5735
XCC1390	tr|Q8PAT7|Q8PAT7_XANCP Uncharacterized protein OS=Xanthomonas campestris pv. campestris (strain ATCC 33913 / NCPPB 528 / LMG 568) GN=XCC1390 PE=4 SV=1	0.0150	2.5823
glgX	tr|Q8P664|Q8P664_XANCP Glycogen operon protein GlgX homolog OS=Xanthomonas campestris pv. campestris (strain ATCC 33913 / NCPPB 528 / LMG 568) GN=glgX PE=4 SV=1	0.0172	2.5898
glgX	sp|A8GKU9|GLGX_SERP5 Glycogen debranching enzyme OS=Serratia proteamaculans (strain 568) GN=glgX PE=3 SV=1	0.0329	2.5979
ML0594	sp|Q49682|Y594_MYCLE UPF0051 protein ML0594 OS=Mycobacterium leprae (strain TN) GN=ML0594 PE=3 SV=1	0.0047	2.5988
XCC3526	tr|Q8P522|Q8P522_XANCP Uncharacterized protein OS=Xanthomonas campestris pv. campestris (strain ATCC 33913 / NCPPB 528 / LMG 568) GN=XCC3526 PE=4 SV=1	0.0239	2.6176
acn	sp|P37032|ACON_LEGPH Aconitate hydratase A OS=Legionella pneumophila subsp. pneumophila (strain Philadelphia 1 / ATCC 33152 / DSM 7513) GN=acn PE=1 SV=1	0.0088	2.6209
gabD1	sp|Q7U2I0|GABD1_MYCBO Succinate-semialdehyde dehydrogenase [NADP(+)] 1 OS=Mycobacterium bovis (strain ATCC BAA-935 / AF2122/97) GN=gabD1 PE=3 SV=2	0.0398	2.6344
lemA	sp|A8AVK0|LEMA_STRGC Protein LemA OS=Streptococcus gordonii (strain Challis / ATCC 35105 / CH1 / DL1 / V288) GN=lemA PE=2 SV=1	0.0047	2.6563
egl	sp|P58599|GUN_RALSO Endoglucanase OS=Ralstonia solanacearum (strain GMI1000) GN=egl PE=3 SV=1	0.0020	2.6572
glgX	sp|Q664I3|GLGX_YERPS Glycogen debranching enzyme OS=Yersinia pseudotuberculosis serotype I (strain IP32953) GN=glgX PE=3 SV=1	0.0235	2.6687

XCV4158	tr|Q3BMX4|Q3BMX4_XANC5 Xanthomonas campestris pv. vesicatoria complete genome OS=Xanthomonas campestris pv. vesicatoria (strain 85-10) GN=XCV4158 PE=4 SV=1	0.0225	2.6745
betA	sp|Q8P5D7|BETA_XANCP Oxygen-dependent choline dehydrogenase OS=Xanthomonas campestris pv. campestris (strain ATCC 33913 / NCPPB 528 / LMG 568) GN=betA PE=3 SV=1	0.0230	2.6750
dnaE	sp|P52022|DPO3A_VIBCH DNA polymerase III subunit alpha OS=Vibrio cholerae serotype O1 (strain ATCC 39315 / El Tor Inaba N16961) GN=dnaE PE=3 SV=3	0.0014	2.6819
glgX	sp|Q32AV4|GLGX_SHIDS Glycogen debranching enzyme OS=Shigella dysenteriae serotype 1 (strain Sd197) GN=glgX PE=3 SV=1	0.0083	2.7114
metX	sp|Q8P8L2|METX_XANCP Homoserine O-acetyltransferase OS=Xanthomonas campestris pv. campestris (strain ATCC 33913 / NCPPB 528 / LMG 568) GN=metX PE=3 SV=1	0.0072	2.7230
XOC_0250	tr|G7TJT7|G7TJT7_XANOB Alpha-amylase family protein OS=Xanthomonas oryzae pv. oryzicola (strain BLS256) GN=XOC_0250 PE=4 SV=1	0.0003	2.7279
XCC2308	tr|Q8P8D4|Q8P8D4_XANCP Uncharacterized protein OS=Xanthomonas campestris pv. campestris (strain ATCC 33913 / NCPPB 528 / LMG 568) GN=XCC2308 PE=4 SV=1	0.0019	2.7470
egl	sp|P17974|GUN_RALSL Endoglucanase OS=Ralstonia solanacearum GN=egl PE=1 SV=2	0.0034	2.7638
NA	sp|P55177|YAG5_STAAU Hydrolase in agr operon OS=Staphylococcus aureus PE=3 SV=1	0.0239	2.7837
Arth_4510	tr|A0AWA9|A0AWA9_ARTS2 CHAP domain containing protein OS=Arthrobacter sp. (strain FB24) GN=Arth_4510 PE=4 SV=1	0.0016	2.8095
glmU	sp|Q8PCZ1|GLMU_XANCP Bifunctional protein GlmU OS=Xanthomonas campestris pv. campestris (strain ATCC 33913 / NCPPB 528 / LMG 568) GN=glmU PE=3 SV=1	0.0042	2.8101
nrdB	sp|Q9KFH7|RIR2_BACHD Ribonucleoside-diphosphate reductase subunit beta OS=Bacillus halodurans (strain ATCC BAA-125 / DSM 18197 / FERM 7344 / JCM 9153 / C-125) GN=nrdB PE=1 SV=1	0.0250	2.8120
ahpC	sp|Q49UT8|AHPC_STAS1 Alkyl hydroperoxide reductase subunit C OS=Staphylococcus saprophyticus subsp. saprophyticus (strain ATCC 15305 / DSM 20229) GN=ahpC PE=3 SV=1	0.0010	2.8153
XCR_3694	tr|G0CF83|G0CF83_XANCA Uncharacterized protein OS=Xanthomonas campestris pv. raphani 756C GN=XCR_3694 PE=4 SV=1	0.0169	2.8237
dsbA	sp|O52376|DSBA_PSESM Thiol:disulfide interchange protein DsbA OS=Pseudomonas syringae pv. tomato (strain DC3000) GN=dsbA PE=3 SV=1	0.0007	2.8327
nrdB	sp|Q9KFH7|RIR2_BACHD Ribonucleoside-diphosphate reductase subunit beta OS=Bacillus halodurans (strain ATCC BAA-125 / DSM 18197 / FERM 7344 / JCM 9153 / C-125) GN=nrdB PE=1 SV=1	0.0002	2.8363
Arth_4510	tr|A0AWA9|A0AWA9_ARTS2 CHAP domain containing protein OS=Arthrobacter sp. (strain FB24) GN=Arth_4510 PE=4 SV=1	0.0002	2.8377
GLX3	sp|Q5AF03|HSP31_CANAL Glyoxalase 3 OS=Candida albicans (strain SC5314 / ATCC MYA-2876) GN=GLX3 PE=1 SV=1	0.0059	2.8518
XCC1390	tr|Q8PAT7|Q8PAT7_XANCP Uncharacterized protein OS=Xanthomonas campestris pv. campestris (strain ATCC 33913 / NCPPB 528 / LMG 568) GN=XCC1390 PE=4 SV=1	0.0289	2.8980
slyD	sp|Q9KNX6|SLYD_VIBCH FKBP-type peptidyl-prolyl cis-trans isomerase SlyD OS=Vibrio cholerae serotype O1 (strain ATCC 39315 / El Tor Inaba N16961) GN=slyD PE=1 SV=1	0.0074	2.9042

oprN	tr|Q8PAN7|Q8PAN7_XANCP Outer membrane protein OS=Xanthomonas campestris pv. campestris (strain ATCC 33913 / NCPPB 528 / LMG 568) GN=oprN PE=4 SV=1	0.0030	2.9145
trpS	sp|Q8P3Z4|SYW_XANCP Tryptophan--tRNA ligase OS=Xanthomonas campestris pv. campestris (strain ATCC 33913 / NCPPB 528 / LMG 568) GN=trpS PE=3 SV=1	0.0016	2.9264
ttgD	sp|Q9KWV5|TTGD_PSEPT Toluene efflux pump periplasmic linker protein TtgD OS=Pseudomonas putida (strain DOT-T1E) GN=ttgD PE=2 SV=2	0.0063	2.9266
XCC3889	tr|Q8P427|Q8P427_XANCP Uncharacterized protein OS=Xanthomonas campestris pv. campestris (strain ATCC 33913 / NCPPB 528 / LMG 568) GN=XCC3889 PE=4 SV=1	0.0023	2.9689
ligD	sp|A0R3R7|LIGD_MYCS2 Multifunctional non-homologous end joining protein LigD OS=Mycobacterium smegmatis (strain ATCC 700084 / mc(2)155) GN=ligD PE=1 SV=2	0.0001	2.9699
amyC	sp|P14899|AMY3_DICT6 Alpha-amylase 3 OS=Dictyoglomus thermophilum (strain ATCC 35947 / DSM 3960 / H-6-12) GN=amyC PE=3 SV=2	0.0028	2.9938
ywbI	sp|P39592|YWBI_BACSU Uncharacterized HTH-type transcriptional regulator YwbI OS=Bacillus subtilis (strain 168) GN=ywbI PE=3 SV=1	0.0021	2.9951
glpK	sp|Q8PDI0|GLPK_XANCP Glycerol kinase OS=Xanthomonas campestris pv. campestris (strain ATCC 33913 / NCPPB 528 / LMG 568) GN=glpK PE=3 SV=1	0.0123	3.0086
XCC3885	tr|Q8P431|Q8P431_XANCP Hydrolase or peptidase OS=Xanthomonas campestris pv. campestris (strain ATCC 33913 / NCPPB 528 / LMG 568) GN=XCC3885 PE=1 SV=1	0.0204	3.0291
sbp	tr|B0RU36|B0RU36_XANCB Sbp protein OS=Xanthomonas campestris pv. campestris (strain B100) GN=sbp PE=4 SV=1	0.0158	3.0380
XCC0105	tr|Q8PE76|Q8PE76_XANCP ATP-dependent DNA ligase OS=Xanthomonas campestris pv. campestris (strain ATCC 33913 / NCPPB 528 / LMG 568) GN=XCC0105 PE=4 SV=1	0.0018	3.0744
lptD	sp|Q8PCE0|LPTD_XANCP LPS-assembly protein LptD OS=Xanthomonas campestris pv. campestris (strain ATCC 33913 / NCPPB 528 / LMG 568) GN=lptD PE=3 SV=1	0.0097	3.0744
glgX	sp|A4WFL4|GLGX_ENT38 Glycogen debranching enzyme OS=Enterobacter sp. (strain 638) GN=glgX PE=3 SV=1	0.0006	3.0766
uup	tr|M4WKN0|M4WKN0_XANCI ATPase component of ABC transporter OS=Xanthomonas citri subsp. citri Aw12879 GN=uup PE=3 SV=1	0.0158	3.0789
gabD1	sp|A0PN13|GABD1_MYCUA Succinate-semialdehyde dehydrogenase [NADP(+)] 1 OS=Mycobacterium ulcerans (strain Agy99) GN=gabD1 PE=3 SV=1	0.0016	3.0998
queF	sp|B0RWU6|QUEF_XANCB NADPH-dependent 7-cyano-7-deazaguanine reductase OS=Xanthomonas campestris pv. campestris (strain B100) GN=queF PE=3 SV=1	0.0004	3.1045
ligD	sp|A0R3R7|LIGD_MYCS2 Multifunctional non-homologous end joining protein LigD OS=Mycobacterium smegmatis (strain ATCC 700084 / mc(2)155) GN=ligD PE=1 SV=2	0.0001	3.1046
cqsS	sp|Q9KM66|CQSS_VIBCH CAI-1 autoinducer sensor kinase/phosphatase CqsS OS=Vibrio cholerae serotype O1 (strain ATCC 39315 / El Tor Inaba N16961) GN=cqsS PE=1 SV=1	0.0029	3.1274

rpsT	sp|Q8PBG9|RS20_XANCP 30S ribosomal protein S20 OS=Xanthomonas campestris pv. campestris (strain ATCC 33913 / NCPPB 528 / LMG 568) GN=rpsT PE=3 SV=1	0.0002	3.1452
adh1	sp|P00332|ADH_SCHPO Alcohol dehydrogenase OS=Schizosaccharomyces pombe (strain 972 / ATCC 24843) GN=adh1 PE=1 SV=2	0.0001	3.3749
XCC3073	tr|Q8P697|Q8P697_XANCP Glutathione transferase OS=Xanthomonas campestris pv. campestris (strain ATCC 33913 / NCPPB 528 / LMG 568) GN=XCC3073 PE=4 SV=1	0.0002	3.4612
yhdF	sp|O07575|YHDF_BACSU Uncharacterized oxidoreductase YhdF OS=Bacillus subtilis (strain 168) GN=yhdF PE=3 SV=1	0.0002	3.5062
yiaA	tr|Q8P498|Q8P498_XANCP Membrane protein OS=Xanthomonas campestris pv. campestris (strain ATCC 33913 / NCPPB 528 / LMG 568) GN=yiaA PE=4 SV=1	0.0000	3.5224
mdtB	sp|C6CAI2|MDTB_DICDC Multidrug resistance protein MdtB OS=Dickeya dadantii (strain Ech703) GN=mdtB PE=3 SV=1	0.0000	3.6821
XC_2759	tr|Q4UT15|Q4UT15_XANC8 Uncharacterized protein OS=Xanthomonas campestris pv. campestris (strain 8004) GN=XC_2759 PE=4 SV=1	0.0000	3.6857
XCR_4088	tr|G0CJ86|G0CJ86_XANCA Oxidoreductase OS=Xanthomonas campestris pv. raphani 756C GN=XCR_4088 PE=3 SV=1	0.0000	3.6950
katE	sp|P95539|CATE_PSEPU Catalase HPII OS=Pseudomonas putida GN=katE PE=3 SV=1	0.0000	3.7319
XCR_0031	tr|G0CGQ6|G0CGQ6_XANCA Peptidase propeptide and ypeb domain protein OS=Xanthomonas campestris pv. raphani 756C GN=XCR_0031 PE=4 SV=1	0.0001	3.7706
bamB	sp|F5ZAY7|BAMB_ALTSS Outer membrane protein assembly factor BamB OS=Alteromonas sp. (strain SN2) GN=bamB PE=3 SV=2	0.0042	3.8313
XCC3924	sp|Q8P3Z3|Y3924_XANCP UPF0337 protein XCC3924 OS=Xanthomonas campestris pv. campestris (strain ATCC 33913 / NCPPB 528 / LMG 568) GN=XCC3924 PE=3 SV=1	0.0001	4.1450
XCC2150	tr|Q8P8T8|Q8P8T8_XANCP Uncharacterized protein OS=Xanthomonas campestris pv. campestris (strain ATCC 33913 / NCPPB 528 / LMG 568) GN=XCC2150 PE=4 SV=1	0.0001	4.1464
MIMI_R526	sp|Q5UQ83|YR526_MIMIV Putative alpha/beta hydrolase R526 OS=Acanthamoeba polyphaga mimivirus GN=MIMI_R526 PE=1 SV=1	0.0000	4.1891
XCC1318	tr|Q8PB08|Q8PB08_XANCP Uncharacterized protein OS=Xanthomonas campestris pv. campestris (strain ATCC 33913 / NCPPB 528 / LMG 568) GN=XCC1318 PE=4 SV=1	0.0000	4.1934
mdtB	sp|A8AEE6|MDTB_CITK8 Multidrug resistance protein MdtB OS=Citrobacter koseri (strain ATCC BAA-895 / CDC 4225-83 / SGSC4696) GN=mdtB PE=3 SV=1	0.0000	4.2346
katE	sp|P95539|CATE_PSEPU Catalase HPII OS=Pseudomonas putida GN=katE PE=3 SV=1	0.0000	4.3452
yhdF	sp|O07575|YHDF_BACSU Uncharacterized oxidoreductase YhdF OS=Bacillus subtilis (strain 168) GN=yhdF PE=3 SV=1	0.0000	4.3609
XCC0134	tr|Q8PE49|Q8PE49_XANCP Trehalose synthase OS=Xanthomonas campestris pv. campestris (strain ATCC 33913 / NCPPB 528 / LMG 568) GN=XCC0134 PE=4 SV=1	0.0001	4.4547
XCR_3876	tr|G0CGM5|G0CGM5_XANCA Cellulase OS=Xanthomonas campestris pv. raphani 756C GN=XCR_3876 PE=3 SV=1	0.0000	4.5112
XCC2020	tr|Q8P946|Q8P946_XANCP Uncharacterized protein OS=Xanthomonas campestris pv. campestris (strain ATCC 33913 / NCPPB 528 / LMG 568) GN=XCC2020 PE=4 SV=1	0.0000	5.0551

CA_C2800	sp|Q97FE0|MCAT_CLOAB Probable manganese catalase OS=Clostridium acetobutylicum (strain ATCC 824 / DSM 792 / JCM 1419 / LMG 5710 / VKM B-1787) GN=CA_C2800 PE=3 SV=1	0.0000	5.5156
**List of up-regulated DEGs in the comparison I3CvsControl**			
Gene	Description	fdr	log2fc
glgZ	tr|B0RMT8|B0RMT8_XANCB Malto-oligosyltrehalose trehalohydrolase OS=Xanthomonas campestris pv. campestris (strain B100) GN=glgZ PE=3 SV=1	0.0283	2.3751
rhaB	sp|C6DJR3|RHAB_PECCP Rhamnulokinase OS=Pectobacterium carotovorum subsp. carotovorum (strain PC1) GN=rhaB PE=3 SV=1	0.0051	2.4490
rplB	sp|Q9PE73|RL2_XYLFA 50S ribosomal protein L2 OS=Xylella fastidiosa (strain 9a5c) GN=rplB PE=3 SV=1	0.0302	2.5690
sodC2	sp|O66602|SODC2_AQUAE Superoxide dismutase [Cu-Zn] 2 OS=Aquifex aeolicus (strain VF5) GN=sodC2 PE=3 SV=1	0.0262	2.5894
macB	sp|Q7ULB5|MACB_RHOBA Macrolide export ATP-binding/permease protein MacB OS=Rhodopirellula baltica (strain SH1) GN=macB PE=3 SV=1	0.0001	2.5995
aroE	sp|Q8P3W6|AROE_XANCP Shikimate dehydrogenase (NADP(+)) OS=Xanthomonas campestris pv. campestris (strain ATCC 33913 / NCPPB 528 / LMG 568) GN=aroE PE=3 SV=1	0.0221	2.6197
speA	sp|Q8P448|SPEA_XANCP Biosynthetic arginine decarboxylase OS=Xanthomonas campestris pv. campestris (strain ATCC 33913 / NCPPB 528 / LMG 568) GN=speA PE=3 SV=2	0.0086	2.6302
rne	tr|Q5GY94|Q5GY94_XANOR Ribonuclease E OS=Xanthomonas oryzae pv. oryzae (strain KACC10331 / KXO85) GN=rne PE=3 SV=1	0.0262	2.6388
leuS	sp|Q8P7J1|SYL_XANCP Leucine--tRNA ligase OS=Xanthomonas campestris pv. campestris (strain ATCC 33913 / NCPPB 528 / LMG 568) GN=leuS PE=3 SV=2	0.0255	2.6440
XCC1489	tr|Q8PAJ4|Q8PAJ4_XANCP Uncharacterized protein OS=Xanthomonas campestris pv. campestris (strain ATCC 33913 / NCPPB 528 / LMG 568) GN=XCC1489 PE=4 SV=1	0.0428	2.6494
XCC4164	tr|Q8P3B2|Q8P3B2_XANCP Uncharacterized protein OS=Xanthomonas campestris pv. campestris (strain ATCC 33913 / NCPPB 528 / LMG 568) GN=XCC4164 PE=4 SV=1	0.0316	2.6574
HI_1056	sp|P71366|T3MH_HAEIN Putative type III restriction-modification system HindVIP enzyme mod OS=Haemophilus influenzae (strain ATCC 51907 / DSM 11121 / KW20 / Rd) GN=HI_1056 PE=3 SV=1	0.0334	2.6771
engXCA	sp|P19487|GUNA_XANCP Major extracellular endoglucanase OS=Xanthomonas campestris pv. campestris (strain ATCC 33913 / NCPPB 528 / LMG 568) GN=engXCA PE=1 SV=2	0.0385	2.7093
ligD	sp|A0R3R7|LIGD_MYCS2 Multifunctional non-homologous end joining protein LigD OS=Mycobacterium smegmatis (strain ATCC 700084 / mc(2)155) GN=ligD PE=1 SV=2	0.0352	2.7345
tolB	sp|B8JD21|TOLB_ANAD2 Protein TolB OS=Anaeromyxobacter dehalogenans (strain 2CP-1 / ATCC BAA-258) GN=tolB PE=3 SV=1	0.0235	2.7755

oar	tr|V7ZCB1|V7ZCB1_9XANT Oar protein OS=Xanthomonas hortorum pv. carotae str. M081 GN=oar PE=4 SV=1	0.0384	2.7951
acsA	sp|Q8P3L1|ACSA_XANCP Acetyl-coenzyme A synthetase OS=Xanthomonas campestris pv. campestris (strain ATCC 33913 / NCPPB 528 / LMG 568) GN=acsA PE=3 SV=1	0.0286	2.7962
malQ	tr|B0RMT9|B0RMT9_XANCB MalQ protein OS=Xanthomonas campestris pv. campestris (strain B100) GN=malQ PE=4 SV=1	0.0177	2.8066
Alvin_0064	sp|P45373|Y064_ALLVD Uncharacterized protein Alvin_0064 OS=Allochromatium vinosum (strain ATCC 17899 / DSM 180 / NBRC 103801 / NCIMB 10441 / D) GN=Alvin_0064 PE=4 SV=1	0.0229	2.8164
secD	tr|Q8P870|Q8P870_XANCP Protein translocase subunit SecD OS=Xanthomonas campestris pv. campestris (strain ATCC 33913 / NCPPB 528 / LMG 568) GN=secD PE=3 SV=1	0.0084	2.8177
dnaN	sp|Q9KVX5|DPO3B_VIBCH DNA polymerase III subunit beta OS=Vibrio cholerae serotype O1 (strain ATCC 39315 / El Tor Inaba N16961) GN=dnaN PE=3 SV=1	0.0298	2.8227
oar	tr|Q8P7N8|Q8P7N8_XANCP Oar protein OS=Xanthomonas campestris pv. campestris (strain ATCC 33913 / NCPPB 528 / LMG 568) GN=oar PE=4 SV=1	0.0239	2.8290
parC	sp|Q1RGX8|PARC_RICBR DNA topoisomerase 4 subunit A OS=Rickettsia bellii (strain RML369-C) GN=parC PE=3 SV=1	0.0324	2.8367
fruA	sp|P23355|PTFBC_XANCP PTS system fructose-specific EIIBC component OS=Xanthomonas campestris pv. campestris (strain ATCC 33913 / NCPPB 528 / LMG 568) GN=fruA PE=1 SV=2	0.0255	2.8552
XACM_1330	tr|G2LXB4|G2LXB4_9XANT Uncharacterized protein OS=Xanthomonas axonopodis pv. citrumelo F1 GN=XACM_1330 PE=4 SV=1	0.0427	2.8646
incC	sp|P07673|INCC2_ECOLX Protein IncC OS=Escherichia coli GN=incC PE=3 SV=1	0.0305	2.8696
XCC3655	tr|Q8P4Q2|Q8P4Q2_XANCP Uncharacterized protein OS=Xanthomonas campestris pv. campestris (strain ATCC 33913 / NCPPB 528 / LMG 568) GN=XCC3655 PE=4 SV=1	0.0021	2.8789
gyrB	sp|O67137|GYRB_AQUAE DNA gyrase subunit B OS=Aquifex aeolicus (strain VF5) GN=gyrB PE=3 SV=1	0.0062	2.8927
asn1	sp|P78753|ASNS_SCHPO Probable asparagine synthetase [glutamine-hydrolyzing] OS=Schizosaccharomyces pombe (strain 972 / ATCC 24843) GN=asn1 PE=1 SV=3	0.0487	2.9087
typA	sp|P57508|TYPA_BUCAI GTP-binding protein TypA/BipA homolog OS=Buchnera aphidicola subsp. Acyrthosiphon pisum (strain APS) GN=typA PE=3 SV=1	0.0248	2.9179
rne	sp|Q8K9J9|RNE_BUCAP Ribonuclease E OS=Buchnera aphidicola subsp. Schizaphis graminum (strain Sg) GN=rne PE=3 SV=1	0.0123	2.9212
secD	sp|O33517|SECD_RHOCB Protein translocase subunit SecD OS=Rhodobacter capsulatus (strain ATCC BAA-309 / NBRC 16581 / SB1003) GN=secD PE=3 SV=1	0.0269	2.9242
pheS	sp|Q8P7Z5|SYFA_XANCP Phenylalanine--tRNA ligase alpha subunit OS=Xanthomonas campestris pv. campestris (strain ATCC 33913 / NCPPB 528 / LMG 568) GN=pheS PE=3 SV=1	0.0206	2.9244
XCC2057	tr|Q8P909|Q8P909_XANCP Phage-related protein OS=Xanthomonas campestris pv. campestris (strain ATCC 33913 / NCPPB 528 / LMG 568) GN=XCC2057 PE=4 SV=1	0.0155	2.9280

parE	sp|Q1RK03|PARE_RICBR DNA topoisomerase 4 subunit B OS=Rickettsia bellii (strain RML369-C) GN=parE PE=3 SV=1	0.0439	2.9280
cysK	sp|O32978|CYSK_MYCLE O-acetylserine sulfhydrylase OS=Mycobacterium leprae (strain TN) GN=cysK PE=3 SV=1	0.0271	2.9319
gltB	sp|Q05755|GLTB_AZOBR Glutamate synthase [NADPH] large chain OS=Azospirillum brasilense GN=gltB PE=1 SV=1	0.0149	2.9418
fabA	sp|Q8PCW9|FABA_XANCP 3-hydroxydecanoyl-[acyl-carrier-protein] dehydratase OS=Xanthomonas campestris pv. campestris (strain ATCC 33913 / NCPPB 528 / LMG 568) GN=fabA PE=3 SV=1	0.0060	2.9521
oar	tr|Q8P7N8|Q8P7N8_XANCP Oar protein OS=Xanthomonas campestris pv. campestris (strain ATCC 33913 / NCPPB 528 / LMG 568) GN=oar PE=4 SV=1	0.0470	2.9523
cysG	sp|Q9PF46|CYSG_XYLFA Siroheme synthase OS=Xylella fastidiosa (strain 9a5c) GN=cysG PE=3 SV=1	0.0106	2.9548
purC	sp|Q3BYD2|PUR7_XANC5 Phosphoribosylaminoimidazole-succinocarboxamide synthase OS=Xanthomonas campestris pv. vesicatoria (strain 85-10) GN=purC PE=3 SV=1	0.0306	2.9561
nrdB	sp|Q9KFH7|RIR2_BACHD Ribonucleoside-diphosphate reductase subunit beta OS=Bacillus halodurans (strain ATCC BAA-125 / DSM 18197 / FERM 7344 / JCM 9153 / C-125) GN=nrdB PE=1 SV=1	0.0136	2.9583
sotB	sp|B1J9Y3|SOTB_PSEPW Probable sugar efflux transporter OS=Pseudomonas putida (strain W619) GN=sotB PE=3 SV=1	0.0295	2.9718
orn	sp|Q8P8S1|ORN_XANCP Oligoribonuclease OS=Xanthomonas campestris pv. campestris (strain ATCC 33913 / NCPPB 528 / LMG 568) GN=orn PE=1 SV=1	0.0169	2.9876
asnS	sp|Q8PAC4|SYN_XANCP Asparagine--tRNA ligase OS=Xanthomonas campestris pv. campestris (strain ATCC 33913 / NCPPB 528 / LMG 568) GN=asnS PE=3 SV=1	0.0416	2.9881
XCC0007	tr|Q8PEG9|Q8PEG9_XANCP Uncharacterized protein OS=Xanthomonas campestris pv. campestris (strain ATCC 33913 / NCPPB 528 / LMG 568) GN=XCC0007 PE=4 SV=1	0.0345	2.9895
dnaB	sp|Q8K932|DNAB_BUCAP Replicative DNA helicase OS=Buchnera aphidicola subsp. Schizaphis graminum (strain Sg) GN=dnaB PE=3 SV=1	0.0088	3.0100
glyS	sp|Q8P3I5|SYGB_XANCP Glycine--tRNA ligase beta subunit OS=Xanthomonas campestris pv. campestris (strain ATCC 33913 / NCPPB 528 / LMG 568) GN=glyS PE=3 SV=1	0.0303	3.0186
XCC3743	tr|Q8P4G5|Q8P4G5_XANCP Uncharacterized protein OS=Xanthomonas campestris pv. campestris (strain ATCC 33913 / NCPPB 528 / LMG 568) GN=XCC3743 PE=4 SV=1	0.0096	3.0381
NGR_a02410	sp|P55577|Y4NA_RHISN Uncharacterized peptidase y4nA OS=Rhizobium sp. (strain NGR234) GN=NGR_a02410 PE=3 SV=1	0.0385	3.0495
XHC_1649	tr|V7ZF87|V7ZF87_9XANT Metallopeptidase OS=Xanthomonas hortorum pv. carotae str. M081 GN=XHC_1649 PE=4 SV=1	0.0173	3.0510
RP373	sp|Q9ZDF6|MAO2_RICPR Probable NADP-dependent malic enzyme OS=Rickettsia prowazekii (strain Madrid E) GN=RP373 PE=3 SV=1	0.0229	3.0682
XC_2759	tr|Q4UT15|Q4UT15_XANC8 Uncharacterized protein OS=Xanthomonas campestris pv. campestris (strain 8004) GN=XC_2759 PE=4 SV=1	0.0248	3.0703
accC	sp|O52058|ACCC_ALLVD Biotin carboxylase OS=Allochromatium vinosum (strain ATCC 17899 / DSM 180 / NBRC 103801 / NCIMB 10441 / D) GN=accC PE=3 SV=2	0.0040	3.0726

NA	sp|Q43914|DLHH_AZOBR Putative carboxymethylenebutenolidase OS=Azospirillum brasilense PE=3 SV=1	0.0016	3.0931
atpA	sp|Q9KNH3|ATPA_VIBCH ATP synthase subunit alpha OS=Vibrio cholerae serotype O1 (strain ATCC 39315 / El Tor Inaba N16961) GN=atpA PE=3 SV=2	0.0339	3.1088
rpsB	sp|Q4USR2|RS2_XANC8 30S ribosomal protein S2 OS=Xanthomonas campestris pv. campestris (strain 8004) GN=rpsB PE=3 SV=1	0.0255	3.1105
btuB	tr|Q8P6U8|Q8P6U8_XANCP TonB-dependent receptor OS=Xanthomonas campestris pv. campestris (strain ATCC 33913 / NCPPB 528 / LMG 568) GN=btuB PE=3 SV=1	0.0057	3.1299
phnA	sp|Q02419|PHNA_STRMU Protein PhnA OS=Streptococcus mutans serotype c (strain ATCC 700610 / UA159) GN=phnA PE=4 SV=2	0.0385	3.1302
der	sp|Q668A3|DER_YERPS GTPase Der OS=Yersinia pseudotuberculosis serotype I (strain IP32953) GN=der PE=3 SV=1	0.0380	3.1315
sbpA	sp|P27366|SUBI_SYNE7 Sulfate-binding protein OS=Synechococcus elongatus (strain PCC 7942) GN=sbpA PE=2 SV=1	0.0428	3.1404
rplM	sp|B4SLE1|RL13_STRM5 50S ribosomal protein L13 OS=Stenotrophomonas maltophilia (strain R551-3) GN=rplM PE=3 SV=1	0.0210	3.1479
secF	tr|G7TBP2|G7TBP2_XANOB Protein-export membrane protein SecF OS=Xanthomonas oryzae pv. oryzicola (strain BLS256) GN=secF PE=3 SV=1	0.0044	3.1539
pdeA	tr|Q8P9A7|Q8P9A7_XANCP C-di-GMP phosphodiesterase A OS=Xanthomonas campestris pv. campestris (strain ATCC 33913 / NCPPB 528 / LMG 568) GN=pdeA PE=4 SV=1	0.0499	3.1591
NA	uncharacterized RNA	0.0352	3.1594
XCC1181	tr|Q8PBE1|Q8PBE1_XANCP Uncharacterized protein OS=Xanthomonas campestris pv. campestris (strain ATCC 33913 / NCPPB 528 / LMG 568) GN=XCC1181 PE=4 SV=1	0.0335	3.1701
glgX	sp|Q664I3|GLGX_YERPS Glycogen debranching enzyme OS=Yersinia pseudotuberculosis serotype I (strain IP32953) GN=glgX PE=3 SV=1	0.0428	3.1987
wxcM	tr|B0RVL6|B0RVL6_XANCB WxcM protein OS=Xanthomonas campestris pv. campestris (strain B100) GN=wxcM PE=4 SV=1	0.0121	3.2128
rpmB	sp|B8GUR2|RL28_THISH 50S ribosomal protein L28 OS=Thioalkalivibrio sulfidiphilus (strain HL-EbGR7) GN=rpmB PE=3 SV=1	0.0013	3.2179
XCC0007	tr|Q8PEG9|Q8PEG9_XANCP Uncharacterized protein OS=Xanthomonas campestris pv. campestris (strain ATCC 33913 / NCPPB 528 / LMG 568) GN=XCC0007 PE=4 SV=1	0.0040	3.2205
ihfB	sp|Q9PAQ8|IHFB_XYLFA Integration host factor subunit beta OS=Xylella fastidiosa (strain 9a5c) GN=ihfB PE=3 SV=2	0.0057	3.2493
XOC_4349	tr|G7TM65|G7TM65_XANOB Transcriptional regulatory protein OS=Xanthomonas oryzae pv. oryzicola (strain BLS256) GN=XOC_4349 PE=4 SV=1	0.0236	3.2597
lrp	sp|P45265|LRP_HAEIN Leucine-responsive regulatory protein OS=Haemophilus influenzae (strain ATCC 51907 / DSM 11121 / KW20 / Rd) GN=lrp PE=3 SV=1	0.0072	3.2867
NlpD	tr|Q5H1V1|Q5H1V1_XANOR Membrane proteins related to metalloendopeptidases OS=Xanthomonas oryzae pv. oryzae (strain KACC10331 / KXO85) GN=NlpD PE=4 SV=1	0.0002	3.2898
dsbA	sp|O52376|DSBA_PSESM Thiol:disulfide interchange protein DsbA OS=Pseudomonas syringae pv. tomato (strain DC3000) GN=dsbA PE=3 SV=1	0.0023	3.2958

aprE	tr|M4WLM8|M4WLM8_XANCI Subtilisin-like serine protease OS=Xanthomonas citri subsp. citri Aw12879 GN=aprE PE=4 SV=1	0.0325	3.3025
malR	sp|P72396|MALR_STRCO HTH-type transcriptional regulator MalR OS=Streptomyces coelicolor (strain ATCC BAA-471 / A3(2) / M145) GN=malR PE=3 SV=2	0.0188	3.3112
radA	sp|Q92F42|RADA_LISIN DNA repair protein RadA homolog OS=Listeria innocua serovar 6a (strain CLIP 11262) GN=radA PE=3 SV=1	0.0039	3.3130
XCR_3876	tr|G0CGM5|G0CGM5_XANCA Cellulase OS=Xanthomonas campestris pv. raphani 756C GN=XCR_3876 PE=3 SV=1	0.0103	3.3348
btuB	sp|Q8DD41|BTUB_VIBVU Vitamin B12 transporter BtuB OS=Vibrio vulnificus (strain CMCP6) GN=btuB PE=3 SV=1	0.0108	3.3489
XCC0449	tr|Q8PD91|Q8PD91_XANCP Lytic enzyme OS=Xanthomonas campestris pv. campestris (strain ATCC 33913 / NCPPB 528 / LMG 568) GN=XCC0449 PE=4 SV=1	0.0332	3.3511
Shewmr4_2217	sp|Q0HI27|NDPA_SHESM Nucleoid-associated protein Shewmr4_2217 OS=Shewanella sp. (strain MR-4) GN=Shewmr4_2217 PE=3 SV=1	0.0008	3.3549
glgX	sp|A8GKU9|GLGX_SERP5 Glycogen debranching enzyme OS=Serratia proteamaculans (strain 568) GN=glgX PE=3 SV=1	0.0419	3.3631
nadE	sp|Q9PC24|NADE_XYLFA Probable glutamine-dependent NAD(+) synthetase OS=Xylella fastidiosa (strain 9a5c) GN=nadE PE=3 SV=1	0.0174	3.3652
oar	tr|M4VWL2|M4VWL2_XANCI Oar protein OS=Xanthomonas citri subsp. citri Aw12879 GN=oar PE=4 SV=1	0.0003	3.3744
XCC3103	tr|Q8P669|Q8P669_XANCP Uncharacterized protein OS=Xanthomonas campestris pv. campestris (strain ATCC 33913 / NCPPB 528 / LMG 568) GN=XCC3103 PE=4 SV=1	0.0060	3.3811
mgtE	tr|Q8P3S9|Q8P3S9_XANCP Magnesium transporter MgtE OS=Xanthomonas campestris pv. campestris (strain ATCC 33913 / NCPPB 528 / LMG 568) GN=mgtE PE=3 SV=1	0.0218	3.3952
treA	sp|Q8P519|TREA_XANCP Periplasmic trehalase OS=Xanthomonas campestris pv. campestris (strain ATCC 33913 / NCPPB 528 / LMG 568) GN=treA PE=3 SV=1	0.0068	3.4056
yhdF	sp|O07575|YHDF_BACSU Uncharacterized oxidoreductase YhdF OS=Bacillus subtilis (strain 168) GN=yhdF PE=3 SV=1	0.0023	3.4130
lly	sp|Q5ZT84|LLY_LEGPH 4-hydroxyphenylpyruvate dioxygenase OS=Legionella pneumophila subsp. pneumophila (strain Philadelphia 1 / ATCC 33152 / DSM 7513) GN=lly PE=1 SV=2	0.0113	3.4284
SPCC13B11.04c	sp|O74540|FADH2_SCHPO Putative S-(hydroxymethyl)glutathione dehydrogenase 2 OS=Schizosaccharomyces pombe (strain 972 / ATCC 24843) GN=SPCC13B11.04c PE=3 SV=2	0.0062	3.4309
NA	sp|P55177|YAG5_STAAU Hydrolase in agr operon OS=Staphylococcus aureus PE=3 SV=1	0.0187	3.4312
pep	tr|B0RNJ4|B0RNJ4_XANCB Pep protein OS=Xanthomonas campestris pv. campestris (strain B100) GN=pep PE=4 SV=1	0.0146	3.4318
tsf	sp|Q87A70|EFTS_XYLFT Elongation factor Ts OS=Xylella fastidiosa (strain Temecula1 / ATCC 700964) GN=tsf PE=3 SV=1	0.0083	3.4567
ubiG	sp|Q8P8H2|UBIG_XANCP Ubiquinone biosynthesis O-methyltransferase OS=Xanthomonas campestris pv. campestris (strain ATCC 33913 / NCPPB 528 / LMG 568) GN=ubiG PE=3 SV=1	0.0137	3.5042
serA	sp|P43885|SERA_HAEIN D-3-phosphoglycerate dehydrogenase OS=Haemophilus influenzae (strain ATCC 51907 / DSM 11121 / KW20 / Rd) GN=serA PE=3 SV=1	0.0223	3.5050

NGR_a02410	sp|P55577|Y4NA_RHISN Uncharacterized peptidase y4nA OS=Rhizobium sp. (strain NGR234) GN=NGR_a02410 PE=3 SV=1	0.0121	3.5104
NA	uncharacterized RNA	0.0277	3.5179
fadB	tr|Q8PB58|Q8PB58_XANCP 3-hydroxyacyl-CoA dehydrogenase OS=Xanthomonas campestris pv. campestris (strain ATCC 33913 / NCPPB 528 / LMG 568) GN=fadB PE=4 SV=1	0.0393	3.5211
cysW	sp|P27370|CYSW_SYNE7 Sulfate transport system permease protein CysW OS=Synechococcus elongatus (strain PCC 7942) GN=cysW PE=2 SV=1	0.0294	3.5684
bfeA	tr|Q8P691|Q8P691_XANCP Ferric enterobactin receptor OS=Xanthomonas campestris pv. campestris (strain ATCC 33913 / NCPPB 528 / LMG 568) GN=bfeA PE=3 SV=1	0.0103	3.5981
atpA	sp|Q3IK48|ATPA_PSEHT ATP synthase subunit alpha OS=Pseudoalteromonas haloplanktis (strain TAC 125) GN=atpA PE=3 SV=1	0.0114	3.5996
katE	sp|P95539|CATE_PSEPU Catalase HPII OS=Pseudomonas putida GN=katE PE=3 SV=1	0.0011	3.6384
mrcA	sp|Q9PGD4|PBPA_XYLFA Penicillin-binding protein 1A OS=Xylella fastidiosa (strain 9a5c) GN=mrcA PE=3 SV=2	0.0187	3.6435
pilA	tr|B0RPQ2|B0RPQ2_XANCB PilA protein OS=Xanthomonas campestris pv. campestris (strain B100) GN=pilA PE=3 SV=1	0.0002	3.6560
rpsT	sp|Q8PBG9|RS20_XANCP 30S ribosomal protein S20 OS=Xanthomonas campestris pv. campestris (strain ATCC 33913 / NCPPB 528 / LMG 568) GN=rpsT PE=3 SV=1	0.0009	3.6806
pilE	tr|Q5H2I1|Q5H2I1_XANOR Pilin OS=Xanthomonas oryzae pv. oryzae (strain KACC10331 / KXO85) GN=pilE PE=3 SV=1	0.0016	3.6825
coaX	sp|Q8P3Y1|COAX_XANCP Type III pantothenate kinase OS=Xanthomonas campestris pv. campestris (strain ATCC 33913 / NCPPB 528 / LMG 568) GN=coaX PE=3 SV=1	0.0000	3.7276
XCAW_00467	tr|M4WBU1|M4WBU1_XANCI Distinct helicase family with a unique C-terminal domain including a metal-binding cysteine cluster OS=Xanthomonas citri subsp. citri Aw12879 GN=XCAW_00467 PE=4 SV=1	0.0414	3.7430
recA	sp|B8GQV3|RECA_THISH Protein RecA OS=Thioalkalivibrio sulfidiphilus (strain HL-EbGR7) GN=recA PE=3 SV=1	0.0049	3.7452
radA	sp|Q9KGG1|RADA_BACHD DNA repair protein RadA homolog OS=Bacillus halodurans (strain ATCC BAA-125 / DSM 18197 / FERM 7344 / JCM 9153 / C-125) GN=radA PE=3 SV=1	0.0012	3.7728
XCC2020	tr|Q8P946|Q8P946_XANCP Uncharacterized protein OS=Xanthomonas campestris pv. campestris (strain ATCC 33913 / NCPPB 528 / LMG 568) GN=XCC2020 PE=4 SV=1	0.0195	3.7930
trpE	sp|P20579|TRPE_PSEPU Anthranilate synthase component 1 OS=Pseudomonas putida GN=trpE PE=3 SV=1	0.0028	3.9119
metB	tr|G7TB12|G7TB12_XANOB Cystathionine gamma-lyase-like protein OS=Xanthomonas oryzae pv. oryzicola (strain BLS256) GN=metB PE=3 SV=1	0.0005	3.9209
CA_C2800	sp|Q97FE0|MCAT_CLOAB Probable manganese catalase OS=Clostridium acetobutylicum (strain ATCC 824 / DSM 792 / JCM 1419 / LMG 5710 / VKM B-1787) GN=CA_C2800 PE=3 SV=1	0.0000	3.9434
recX	sp|Q8P9X1|RECX_XANCP Regulatory protein RecX OS=Xanthomonas campestris pv. campestris (strain ATCC 33913 / NCPPB 528 / LMG 568) GN=recX PE=1 SV=1	0.0002	3.9438

argG	sp|Q8P8J4|ASSY_XANCP Argininosuccinate synthase OS=Xanthomonas campestris pv. campestris (strain ATCC 33913 / NCPPB 528 / LMG 568) GN=argG PE=3 SV=1	0.0000	4.0627
yliI	tr|Q8P497|Q8P497_XANCP Dehydrogenase OS=Xanthomonas campestris pv. campestris (strain ATCC 33913 / NCPPB 528 / LMG 568) GN=yliI PE=4 SV=1	0.0340	4.0633
rhlE	tr|Q8PDB5|Q8PDB5_XANCP ATP-dependent RNA helicase OS=Xanthomonas campestris pv. campestris (strain ATCC 33913 / NCPPB 528 / LMG 568) GN=rhlE PE=3 SV=1	0.0050	4.0714
yeiG	sp|B1LKQ1|SFGH2_ECOSM S-formylglutathione hydrolase YeiG OS=Escherichia coli (strain SMS-3-5 / SECEC) GN=yeiG PE=3 SV=1	0.0499	4.0967
uup	tr|M4WKN0|M4WKN0_XANCI ATPase component of ABC transporter OS=Xanthomonas citri subsp. citri Aw12879 GN=uup PE=3 SV=1	0.0016	4.1247
metB	sp|P46807|METB_MYCLE Cystathionine gamma-synthase OS=Mycobacterium leprae (strain TN) GN=metB PE=3 SV=1	0.0005	4.1673
GSU0804	sp|Q74F05|NQOR_GEOSL NAD(P)H dehydrogenase (quinone) OS=Geobacter sulfurreducens (strain ATCC 51573 / DSM 12127 / PCA) GN=GSU0804 PE=3 SV=1	0.0026	4.1860
argB	sp|Q8P8J6|ARGB_XANCP Acetylglutamate kinase OS=Xanthomonas campestris pv. campestris (strain ATCC 33913 / NCPPB 528 / LMG 568) GN=argB PE=3 SV=2	0.0006	4.4444
prs	sp|A6W1C7|KPRS_MARMS Ribose-phosphate pyrophosphokinase OS=Marinomonas sp. (strain MWYL1) GN=prs PE=3 SV=1	0.0264	4.4639
argG	sp|Q8P8J4|ASSY_XANCP Argininosuccinate synthase OS=Xanthomonas campestris pv. campestris (strain ATCC 33913 / NCPPB 528 / LMG 568) GN=argG PE=3 SV=1	0.0004	4.4897
XCC1390	tr|Q8PAT7|Q8PAT7_XANCP Uncharacterized protein OS=Xanthomonas campestris pv. campestris (strain ATCC 33913 / NCPPB 528 / LMG 568) GN=XCC1390 PE=4 SV=1	0.0080	4.5211
XCC0134	tr|Q8PE49|Q8PE49_XANCP Trehalose synthase OS=Xanthomonas campestris pv. campestris (strain ATCC 33913 / NCPPB 528 / LMG 568) GN=XCC0134 PE=4 SV=1	0.0018	4.5350
metB	sp|P46807|METB_MYCLE Cystathionine gamma-synthase OS=Mycobacterium leprae (strain TN) GN=metB PE=3 SV=1	0.0000	4.7209
gumJ	tr|Q8P805|Q8P805_XANCP GumJ protein OS=Xanthomonas campestris pv. campestris (strain ATCC 33913 / NCPPB 528 / LMG 568) GN=gumJ PE=4 SV=1	0.0164	4.8616
XCC0335	tr|Q7CLW1|Q7CLW1_XANCP Uncharacterized protein OS=Xanthomonas campestris pv. campestris (strain ATCC 33913 / NCPPB 528 / LMG 568) GN=XCC0335 PE=4 SV=1	0.0013	5.0873
sbp	tr|B0RU36|B0RU36_XANCB Sbp protein OS=Xanthomonas campestris pv. campestris (strain B100) GN=sbp PE=4 SV=1	0.0000	5.0991
XCC2730	tr|Q8P783|Q8P783_XANCP Alcohol dehydrogenase OS=Xanthomonas campestris pv. campestris (strain ATCC 33913 / NCPPB 528 / LMG 568) GN=XCC2730 PE=4 SV=1	0.0001	inf
