# Supplementary material for: Transcriptomic Reprograming of Xanthomonas campestris pv. campestris after Treatment with Hydrolytic Products Derived from Glucosinolates
Source: Plants (Basel). 2021 Aug 11;10(8):1656. doi: 10.3390/plants10081656 (PMC8400333; doi:10.3390/plants10081656)
Supplement: Supplementary file 1 [file plants-10-01656-s001.zip › supplementary/Table S2.pdf]

**List of down-regulated DEGs in the comparison AITCvsControl**			
Gene	Description	fdr	log2fc
cydA	tr|Q8P8K8|Q8P8K8_XANCP Cytochrome D ubiquinol oxidase subunit I OS=Xanthomonas campestris pv. campestris (strain ATCC 33913 / NCPPB 528 / LMG 568) GN=cydA PE=4 SV=1	0.0001	-2.4646
fprB	sp|O33064|FPRB_MYCLE Probable ferredoxin/ferredoxin--NADP reductase OS=Mycobacterium leprae (strain TN) GN=fprB PE=3 SV=1	0.0001	-2.3363
dnaK	sp|B2GGP0|DNAK_KOCRD Chaperone protein DnaK OS=Kocuria rhizophila (strain ATCC 9341 / DSM 348 / NBRC 103217 / DC2201) GN=dnaK PE=3 SV=1	0.0001	-2.1019
sir	sp|Q7TYP6|SIR_MYCBO Sulfite reductase [ferredoxin] OS=Mycobacterium bovis (strain ATCC BAA-935 / AF2122/97) GN=sir PE=3 SV=2	0.0005	-2.0521
sir	sp|Q7TYP6|SIR_MYCBO Sulfite reductase [ferredoxin] OS=Mycobacterium bovis (strain ATCC BAA-935 / AF2122/97) GN=sir PE=3 SV=2	0.0007	-1.9709
sir	sp|Q7TYP6|SIR_MYCBO Sulfite reductase [ferredoxin] OS=Mycobacterium bovis (strain ATCC BAA-935 / AF2122/97) GN=sir PE=3 SV=2	0.0006	-1.9579
sir	sp|Q7TYP6|SIR_MYCBO Sulfite reductase [ferredoxin] OS=Mycobacterium bovis (strain ATCC BAA-935 / AF2122/97) GN=sir PE=3 SV=2	0.0008	-1.9403
dnaK	sp|B2GGP0|DNAK_KOCRD Chaperone protein DnaK OS=Kocuria rhizophila (strain ATCC 9341 / DSM 348 / NBRC 103217 / DC2201) GN=dnaK PE=3 SV=1	0.0028	-1.6027
gabT	sp|P40829|GABT_MYCLE 4-aminobutyrate aminotransferase OS=Mycobacterium leprae (strain TN) GN=gabT PE=3 SV=1	0.0016	-1.4717
araG	sp|Q9K7C3|ARAG_BACHD L-arabinose transport ATP-binding protein AraG OS=Bacillus halodurans (strain ATCC BAA-125 / DSM 18197 / FERM 7344 / JCM 9153 / C-125) GN=araG PE=3 SV=1	0.0045	-1.3665
pstS	sp|Q9PBK3|PSTS_XYLFA Phosphate-binding protein PstS OS=Xylella fastidiosa (strain 9a5c) GN=pstS PE=3 SV=1	0.0074	-1.2154
fadA	sp|Q5E8X7|FADA_VIBF1 3-ketoacyl-CoA thiolase OS=Vibrio fischeri (strain ATCC 700601 / ES114) GN=fadA PE=3 SV=2	0.0019	-1.1740
yhdF	sp|O07575|YHDF_BACSU Uncharacterized oxidoreductase YhdF OS=Bacillus subtilis (strain 168) GN=yhdF PE=3 SV=1	0.0007	-1.1377
chvE	sp|P25548|CHVE_AGRT5 Multiple sugar-binding periplasmic receptor ChvE OS=Agrobacterium tumefaciens (strain C58 / ATCC 33970) GN=chvE PE=1 SV=2	0.0000	-1.0631
sir	sp|Q7TYP6|SIR_MYCBO Sulfite reductase [ferredoxin] OS=Mycobacterium bovis (strain ATCC BAA-935 / AF2122/97) GN=sir PE=3 SV=2	0.0045	-1.0589
SPCC965.06	sp|O59826|KCAB_SCHPO Putative voltage-gated potassium channel subunit beta OS=Schizosaccharomyces pombe (strain 972 / ATCC 24843) GN=SPCC965.06 PE=3 SV=1	0.0014	-1.0233

cydA	tr|Q8P8K8|Q8P8K8_XANCP Cytochrome D ubiquinol oxidase subunit I OS=Xanthomonas campestris pv. campestris (strain ATCC 33913 / NCPPB 528 / LMG 568) GN=cydA PE=4 SV=1	0.0026	-1.0130
hpaH	sp|A4IT51|HPAH_GEOTN Anthranilate 3-monooxygenase oxygenase component OS=Geobacillus thermodenitrificans (strain NG80-2) GN=hpaH PE=1 SV=1	0.0010	-0.9880
**List of down-regulated DEGs in the comparison I3CvsControl**			
Gene	Description	fdr	log2fc
P	sp|P17394|DPOL_HBVB1 Protein P OS=Hepatitis B virus genotype B1 subtype adw (isolate Japan/pJDW233/1988) GN=P PE=3 SV=1	0.0013	-9.0488
P	sp|P03158|DPOL_HBVA2 Protein P OS=Hepatitis B virus genotype A2 subtype adw (isolate Japan/Nishioka/1983) GN=P PE=3 SV=2	0.0043	-7.7522
rpsN	sp|A0K1X4|RS14_ARTS2 30S ribosomal protein S14 OS=Arthrobacter sp. (strain FB24) GN=rpsN PE=3 SV=1	0.0000	-3.3717
rpsE	sp|A0JZ68|RS5_ARTS2 30S ribosomal protein S5 OS=Arthrobacter sp. (strain FB24) GN=rpsE PE=3 SV=1	0.0000	-3.3329
glnA	sp|P15106|GLNA_STRCO Glutamine synthetase OS=Streptomyces coelicolor (strain ATCC BAA-471 / A3(2) / M145) GN=glnA PE=2 SV=1	0.0000	-3.1903
rplM	sp|A9WMG0|RL13_RENSM 50S ribosomal protein L13 OS=Renibacterium salmoninarum (strain ATCC 33209 / DSM 20767 / JCM 11484 / NBRC 15589 / NCIMB 2235) GN=rplM PE=3 SV=1	0.0000	-3.0214
sdhA	sp|Q1RHB9|SDHA_RICBR Succinate dehydrogenase flavoprotein subunit OS=Rickettsia bellii (strain RML369-C) GN=sdhA PE=3 SV=1	0.0000	-3.0194
fusA	sp|Q0SFF3|EFG_RHOJR Elongation factor G OS=Rhodococcus jostii (strain RHA1) GN=fusA PE=3 SV=1	0.0000	-2.9591
fusA	sp|Q0SFF3|EFG_RHOJR Elongation factor G OS=Rhodococcus jostii (strain RHA1) GN=fusA PE=3 SV=1	0.0000	-2.9409
ftsX	sp|O32882|FTSX_MYCLE Cell division protein FtsX OS=Mycobacterium leprae (strain TN) GN=ftsX PE=3 SV=2	0.0000	-2.9011
fusA	sp|Q0SFF3|EFG_RHOJR Elongation factor G OS=Rhodococcus jostii (strain RHA1) GN=fusA PE=3 SV=1	0.0000	-2.9003
sdhA	sp|Q1RHB9|SDHA_RICBR Succinate dehydrogenase flavoprotein subunit OS=Rickettsia bellii (strain RML369-C) GN=sdhA PE=3 SV=1	0.0000	-2.8587
atpA	sp|A0JY66|ATPA_ARTS2 ATP synthase subunit alpha OS=Arthrobacter sp. (strain FB24) GN=atpA PE=3 SV=2	0.0000	-2.7353
atpA	sp|A0JY66|ATPA_ARTS2 ATP synthase subunit alpha OS=Arthrobacter sp. (strain FB24) GN=atpA PE=3 SV=2	0.0000	-2.6966

SAU060112_90002	tr|A0A090M192|A0A090M192_STAAU Putative cytoplasmic protein OS=Staphylococcus aureus GN=SAU060112_90002 PE=4 SV=1	0.0000	-2.6409
HMPREF1043_0706	tr|I0S7Z1|I0S7Z1_STRAP Uncharacterized protein OS=Streptococcus anginosus subsp. whileyi CCUG 39159 GN=HMPREF1043_0706 PE=4 SV=1	0.0000	-2.6225
rpsN	sp|A0K1X4|RS14_ARTS2 30S ribosomal protein S14 OS=Arthrobacter sp. (strain FB24) GN=rpsN PE=3 SV=1	0.0000	-2.6123
ctaD1	sp|Q9X813|COX1A_STRCO Probable cytochrome c oxidase subunit 1-alpha OS=Streptomyces coelicolor (strain ATCC BAA-471 / A3(2) / M145) GN=ctaD1 PE=3 SV=1	0.0000	-2.6054
hisC	sp|Q47QS8|HIS8_THEFY Histidinol-phosphate aminotransferase OS=Thermobifida fusca (strain YX) GN=hisC PE=3 SV=1	0.0000	-2.5716
pyk	sp|Q8FP04|KPYK_COREF Pyruvate kinase OS=Corynebacterium efficiens (strain DSM 44549 / YS-314 / AJ 12310 / JCM 11189 / NBRC 100395) GN=pyk PE=3 SV=2	0.0000	-2.4520
sucC	sp|B8HDC2|SUCC_ARTCA Succinyl-CoA ligase [ADP-forming] subunit beta OS=Arthrobacter chlorophenolicus (strain ATCC 700700 / DSM 12829 / JCM 12360 / NCIMB 13794 / A6) GN=sucC PE=3 SV=1	0.0000	-2.3906
sucC	sp|A9WKU9|SUCC_RENSM Succinyl-CoA ligase [ADP-forming] subunit beta OS=Renibacterium salmoninarum (strain ATCC 33209 / DSM 20767 / JCM 11484 / NBRC 15589 / NCIMB 2235) GN=sucC PE=3 SV=1	0.0000	-2.3624
sucC	sp|A9WKU9|SUCC_RENSM Succinyl-CoA ligase [ADP-forming] subunit beta OS=Renibacterium salmoninarum (strain ATCC 33209 / DSM 20767 / JCM 11484 / NBRC 15589 / NCIMB 2235) GN=sucC PE=3 SV=1	0.0000	-2.3512
sucC	sp|A9WKU9|SUCC_RENSM Succinyl-CoA ligase [ADP-forming] subunit beta OS=Renibacterium salmoninarum (strain ATCC 33209 / DSM 20767 / JCM 11484 / NBRC 15589 / NCIMB 2235) GN=sucC PE=3 SV=1	0.0000	-2.3109
rpsB	sp|A0JUP1|RS2_ARTS2 30S ribosomal protein S2 OS=Arthrobacter sp. (strain FB24) GN=rpsB PE=3 SV=1	0.0000	-2.3073
rpsB	sp|A0JUP1|RS2_ARTS2 30S ribosomal protein S2 OS=Arthrobacter sp. (strain FB24) GN=rpsB PE=3 SV=1	0.0000	-2.2859
sucC	sp|A9WKU9|SUCC_RENSM Succinyl-CoA ligase [ADP-forming] subunit beta OS=Renibacterium salmoninarum (strain ATCC 33209 / DSM 20767 / JCM 11484 / NBRC 15589 / NCIMB 2235) GN=sucC PE=3 SV=1	0.0000	-2.2774
rpsB	sp|A0JUP1|RS2_ARTS2 30S ribosomal protein S2 OS=Arthrobacter sp. (strain FB24) GN=rpsB PE=3 SV=1	0.0000	-2.2742
rpsB	sp|A0JUP1|RS2_ARTS2 30S ribosomal protein S2 OS=Arthrobacter sp. (strain FB24) GN=rpsB PE=3 SV=1	0.0000	-2.2455
rpsB	sp|A0JUP1|RS2_ARTS2 30S ribosomal protein S2 OS=Arthrobacter sp. (strain FB24) GN=rpsB PE=3 SV=1	0.0000	-2.2213
rpsB	sp|A0JUP1|RS2_ARTS2 30S ribosomal protein S2 OS=Arthrobacter sp. (strain FB24) GN=rpsB PE=3 SV=1	0.0000	-2.2208
rpsB	sp|A0JUP1|RS2_ARTS2 30S ribosomal protein S2 OS=Arthrobacter sp. (strain FB24) GN=rpsB PE=3 SV=1	0.0000	-2.1976
atpA	sp|A0JY66|ATPA_ARTS2 ATP synthase subunit alpha OS=Arthrobacter sp. (strain FB24) GN=atpA PE=3 SV=2	0.0000	-2.1755
atpA	sp|A0JY66|ATPA_ARTS2 ATP synthase subunit alpha OS=Arthrobacter sp. (strain FB24) GN=atpA PE=3 SV=2	0.0000	-2.1632

atpA	sp|A0JY66|ATPA_ARTS2 ATP synthase subunit alpha OS=Arthrobacter sp. (strain FB24) GN=atpA PE=3 SV=2	0.0000	-2.1625
qcrB	sp|Q9X806|QCRB_STRCO Ubiquinol-cytochrome c reductase cytochrome b subunit OS=Streptomyces coelicolor (strain ATCC BAA-471 / A3(2) / M145) GN=qcrB PE=3 SV=1	0.0000	-2.1527
atpA	sp|A0JY66|ATPA_ARTS2 ATP synthase subunit alpha OS=Arthrobacter sp. (strain FB24) GN=atpA PE=3 SV=2	0.0000	-2.1448
fprB	sp|O33064|FPRB_MYCLE Probable ferredoxin/ferredoxin--NADP reductase OS=Mycobacterium leprae (strain TN) GN=fprB PE=3 SV=1	0.0000	-2.1381
atpA	sp|A0JY66|ATPA_ARTS2 ATP synthase subunit alpha OS=Arthrobacter sp. (strain FB24) GN=atpA PE=3 SV=2	0.0000	-2.1248
rpsB	sp|A0JUP1|RS2_ARTS2 30S ribosomal protein S2 OS=Arthrobacter sp. (strain FB24) GN=rpsB PE=3 SV=1	0.0000	-2.1173
atpA	sp|A0JY66|ATPA_ARTS2 ATP synthase subunit alpha OS=Arthrobacter sp. (strain FB24) GN=atpA PE=3 SV=2	0.0000	-2.1150
rpsB	sp|A0JUP1|RS2_ARTS2 30S ribosomal protein S2 OS=Arthrobacter sp. (strain FB24) GN=rpsB PE=3 SV=1	0.0000	-2.0897
dnaK	sp|B2GGP0|DNAK_KOCRD Chaperone protein DnaK OS=Kocuria rhizophila (strain ATCC 9341 / DSM 348 / NBRC 103217 / DC2201) GN=dnaK PE=3 SV=1	0.0000	-2.0796
atpA	sp|A0JY66|ATPA_ARTS2 ATP synthase subunit alpha OS=Arthrobacter sp. (strain FB24) GN=atpA PE=3 SV=2	0.0000	-2.0762
atpA	sp|A0JY66|ATPA_ARTS2 ATP synthase subunit alpha OS=Arthrobacter sp. (strain FB24) GN=atpA PE=3 SV=2	0.0000	-2.0761
typA	sp|P57508|TYPA_BUCAI GTP-binding protein TypA/BipA homolog OS=Buchnera aphidicola subsp. Acyrthosiphon pisum (strain APS) GN=typA PE=3 SV=1	0.0000	-2.0510
rpsB	sp|A0JUP1|RS2_ARTS2 30S ribosomal protein S2 OS=Arthrobacter sp. (strain FB24) GN=rpsB PE=3 SV=1	0.0000	-2.0425
cheB2	sp|Q6LTM2|CHEB2_PHOPR Chemotaxis response regulator protein-glutamate methylesterase 2 OS=Photobacterium profundum GN=cheB2 PE=3 SV=1	0.0000	-2.0207
rpsB	sp|A0JUP1|RS2_ARTS2 30S ribosomal protein S2 OS=Arthrobacter sp. (strain FB24) GN=rpsB PE=3 SV=1	0.0000	-2.0196
typA	sp|P57508|TYPA_BUCAI GTP-binding protein TypA/BipA homolog OS=Buchnera aphidicola subsp. Acyrthosiphon pisum (strain APS) GN=typA PE=3 SV=1	0.0000	-2.0067
nrdE1	sp|P0CG99|RIR1A_MYCS2 Ribonucleoside-diphosphate reductase subunit alpha 1 OS=Mycobacterium smegmatis (strain ATCC 700084 / mc(2)155) GN=nrdE1 PE=1 SV=1	0.0000	-2.0020
rplM	sp|A9WMG0|RL13_RENSM 50S ribosomal protein L13 OS=Renibacterium salmoninarum (strain ATCC 33209 / DSM 20767 / JCM 11484 / NBRC 15589 / NCIMB 2235) GN=rplM PE=3 SV=1	0.0000	-1.9943
typA	sp|P57508|TYPA_BUCAI GTP-binding protein TypA/BipA homolog OS=Buchnera aphidicola subsp. Acyrthosiphon pisum (strain APS) GN=typA PE=3 SV=1	0.0000	-1.9914
rpsB	sp|A0JUP1|RS2_ARTS2 30S ribosomal protein S2 OS=Arthrobacter sp. (strain FB24) GN=rpsB PE=3 SV=1	0.0000	-1.9896

typA	sp|P57508|TYPA_BUCAI GTP-binding protein TypA/BipA homolog OS=Buchnera aphidicola subsp. Acyrthosiphon pisum (strain APS) GN=typA PE=3 SV=1	0.0000	-1.9887
NA	sp|Q51945|TTUC_PSEPU Tartrate dehydrogenase/decarboxylase OS=Pseudomonas putida PE=1 SV=3	0.0000	-1.9589
typA	sp|P57508|TYPA_BUCAI GTP-binding protein TypA/BipA homolog OS=Buchnera aphidicola subsp. Acyrthosiphon pisum (strain APS) GN=typA PE=3 SV=1	0.0000	-1.9345
araG	sp|Q9K7C3|ARAG_BACHD L-arabinose transport ATP-binding protein AraG OS=Bacillus halodurans (strain ATCC BAA-125 / DSM 18197 / FERM 7344 / JCM 9153 / C-125) GN=araG PE=3 SV=1	0.0000	-1.9199
Mb2838c	sp|P59800|TRA9_MYCBO Putative transposase for insertion sequence element IS986/IS6110 OS=Mycobacterium bovis (strain ATCC BAA-935 / AF2122/97) GN=Mb2838c PE=3 SV=1	0.0000	-1.8907
Achl_0442	tr|B8HAI0|B8HAI0_ARTCA Tartrate dehydrogenase OS=Arthrobacter chlorophenolicus (strain ATCC 700700 / DSM 12829 / JCM 12360 / NCIMB 13794 / A6) GN=Achl_0442 PE=3 SV=1	0.0001	-1.8889
YLR154C-G	sp|Q3E813|YL154_YEAST Uncharacterized protein YLR154C-G OS=Saccharomyces cerevisiae (strain ATCC 204508 / S288c) GN=YLR154C-G PE=2 SV=1	0.0023	-1.8858
parB	sp|Q50201|PARB_MYCLE Probable chromosome-partitioning protein ParB OS=Mycobacterium leprae (strain TN) GN=parB PE=3 SV=1	0.0000	-1.8738
infB	sp|A9WPV8|IF2_RENSM Translation initiation factor IF-2 OS=Renibacterium salmoninarum (strain ATCC 33209 / DSM 20767 / JCM 11484 / NBRC 15589 / NCIMB 2235) GN=infB PE=3 SV=1	0.0000	-1.8648
napA	sp|P81186|NAPA_DESDA Periplasmic nitrate reductase OS=Desulfovibrio desulfuricans (strain ATCC 27774 / DSM 6949) GN=napA PE=1 SV=2	0.0000	-1.8613
groL2	sp|A9WN14|CH602_RENSM 60 kDa chaperonin 2 OS=Renibacterium salmoninarum (strain ATCC 33209 / DSM 20767 / JCM 11484 / NBRC 15589 / NCIMB 2235) GN=groL2 PE=3 SV=1	0.0000	-1.8392
rplI	sp|A0K2H2|RL9_ARTS2 50S ribosomal protein L9 OS=Arthrobacter sp. (strain FB24) GN=rplI PE=3 SV=1	0.0000	-1.8104
sir	sp|Q7TYP6|SIR_MYCBO Sulfite reductase [ferredoxin] OS=Mycobacterium bovis (strain ATCC BAA-935 / AF2122/97) GN=sir PE=3 SV=2	0.0000	-1.7714
sir	sp|Q7TYP6|SIR_MYCBO Sulfite reductase [ferredoxin] OS=Mycobacterium bovis (strain ATCC BAA-935 / AF2122/97) GN=sir PE=3 SV=2	0.0000	-1.7701
cynT	sp|A0R566|CYNT_MYCS2 Carbonic anhydrase OS=Mycobacterium smegmatis (strain ATCC 700084 / mc(2)155) GN=cynT PE=1 SV=1	0.0000	-1.7482
sir	sp|Q7TYP6|SIR_MYCBO Sulfite reductase [ferredoxin] OS=Mycobacterium bovis (strain ATCC BAA-935 / AF2122/97) GN=sir PE=3 SV=2	0.0000	-1.7477

pepA	sp|C5CCM4|AMPA_MICLC Probable cytosol aminopeptidase OS=Micrococcus luteus (strain ATCC 4698 / DSM 20030 / JCM 1464 / NBRC 3333 / NCIMB 9278 / NCTC 2665 / VKM Ac-2230) GN=pepA PE=3 SV=1	0.0000	-1.7411
sir	sp|Q7TYP6|SIR_MYCBO Sulfite reductase [ferredoxin] OS=Mycobacterium bovis (strain ATCC BAA-935 / AF2122/97) GN=sir PE=3 SV=2	0.0000	-1.7411
sepF	sp|A0JVA0|SEPF_ARTS2 Cell division protein SepF OS=Arthrobacter sp. (strain FB24) GN=sepF PE=3 SV=1	0.0000	-1.7358
rplA	sp|B8HD24|RL1_ARTCA 50S ribosomal protein L1 OS=Arthrobacter chlorophenolicus (strain ATCC 700700 / DSM 12829 / JCM 12360 / NCIMB 13794 / A6) GN=rplA PE=3 SV=1	0.0000	-1.7305
atpA	sp|A0JY66|ATPA_ARTS2 ATP synthase subunit alpha OS=Arthrobacter sp. (strain FB24) GN=atpA PE=3 SV=2	0.0000	-1.7262
atpA	sp|A0JY66|ATPA_ARTS2 ATP synthase subunit alpha OS=Arthrobacter sp. (strain FB24) GN=atpA PE=3 SV=2	0.0000	-1.7261
mqo	sp|B8HAT7|MQO_ARTCA Probable malate:quinone oxidoreductase OS=Arthrobacter chlorophenolicus (strain ATCC 700700 / DSM 12829 / JCM 12360 / NCIMB 13794 / A6) GN=mqo PE=3 SV=1	0.0000	-1.7064
rplA	sp|B8HD24|RL1_ARTCA 50S ribosomal protein L1 OS=Arthrobacter chlorophenolicus (strain ATCC 700700 / DSM 12829 / JCM 12360 / NCIMB 13794 / A6) GN=rplA PE=3 SV=1	0.0000	-1.7005
whiB	sp|Q7AKN0|WHIB_STRCO Transcriptional regulator WhiB OS=Streptomyces coelicolor (strain ATCC BAA-471 / A3(2) / M145) GN=whiB PE=2 SV=1	0.0000	-1.6981
hisA	sp|A9WQA3|HIS4_RENSM 1-(5-phosphoribosyl)-5-[(5-phosphoribosylamino)methylideneamino] imidazole-4-carboxamide isomerase OS=Renibacterium salmoninarum (strain ATCC 33209 / DSM 20767 / JCM 11484 / NBRC 15589 / NCIMB 2235) GN=hisA PE=3 SV=1	0.0000	-1.6876
dnaK	sp|B2GGP0|DNAK_KOCRD Chaperone protein DnaK OS=Kocuria rhizophila (strain ATCC 9341 / DSM 348 / NBRC 103217 / DC2201) GN=dnaK PE=3 SV=1	0.0000	-1.6827
pepA	sp|C5CCM4|AMPA_MICLC Probable cytosol aminopeptidase OS=Micrococcus luteus (strain ATCC 4698 / DSM 20030 / JCM 1464 / NBRC 3333 / NCIMB 9278 / NCTC 2665 / VKM Ac-2230) GN=pepA PE=3 SV=1	0.0000	-1.6784
atpA	sp|A0JY66|ATPA_ARTS2 ATP synthase subunit alpha OS=Arthrobacter sp. (strain FB24) GN=atpA PE=3 SV=2	0.0000	-1.6730
hisA	sp|A9WQA3|HIS4_RENSM 1-(5-phosphoribosyl)-5-[(5-phosphoribosylamino)methylideneamino] imidazole-4-carboxamide isomerase OS=Renibacterium salmoninarum (strain ATCC 33209 / DSM 20767 / JCM 11484 / NBRC 15589 / NCIMB 2235) GN=hisA PE=3 SV=1	0.0000	-1.6615
NA	uncharacterized RNA	0.0000	-1.6613
atpA	sp|A0JY66|ATPA_ARTS2 ATP synthase subunit alpha OS=Arthrobacter sp. (strain FB24) GN=atpA PE=3 SV=2	0.0000	-1.6506
atpA	sp|A0JY66|ATPA_ARTS2 ATP synthase subunit alpha OS=Arthrobacter sp. (strain FB24) GN=atpA PE=3 SV=2	0.0000	-1.6458

hisA	sp|A9WQA3|HIS4_RENSM 1-(5-phosphoribosyl)-5-[(5-phosphoribosylamino)methylideneamino] imidazole-4-carboxamide isomerase OS=Renibacterium salmoninarum (strain ATCC 33209 / DSM 20767 / JCM 11484 / NBRC 15589 / NCIMB 2235) GN=hisA PE=3 SV=1	0.0000	-1.6374
sdhA	sp|Q1RHB9|SDHA_RICBR Succinate dehydrogenase flavoprotein subunit OS=Rickettsia bellii (strain RML369-C) GN=sdhA PE=3 SV=1	0.0000	-1.6303
tkt	sp|P46708|TKT_MYCLE Transketolase OS=Mycobacterium leprae (strain TN) GN=tkt PE=3 SV=1	0.0000	-1.6291
cin	sp|P21703|CIN_BPP7 DNA-invertase OS=Enterobacteria phage P7 GN=cin PE=3 SV=1	0.0000	-1.6233
atpA	sp|A0JY66|ATPA_ARTS2 ATP synthase subunit alpha OS=Arthrobacter sp. (strain FB24) GN=atpA PE=3 SV=2	0.0000	-1.6224
hisA	sp|A9WQA3|HIS4_RENSM 1-(5-phosphoribosyl)-5-[(5-phosphoribosylamino)methylideneamino] imidazole-4-carboxamide isomerase OS=Renibacterium salmoninarum (strain ATCC 33209 / DSM 20767 / JCM 11484 / NBRC 15589 / NCIMB 2235) GN=hisA PE=3 SV=1	0.0000	-1.6102
atpA	sp|A0JY66|ATPA_ARTS2 ATP synthase subunit alpha OS=Arthrobacter sp. (strain FB24) GN=atpA PE=3 SV=2	0.0000	-1.6062
mrcA	sp|O66874|PBPA_AQUAE Penicillin-binding protein 1A OS=Aquifex aeolicus (strain VF5) GN=mrcA PE=1 SV=1	0.0000	-1.6058
rplA	sp|B8HD24|RL1_ARTCA 50S ribosomal protein L1 OS=Arthrobacter chlorophenolicus (strain ATCC 700700 / DSM 12829 / JCM 12360 / NCIMB 13794 / A6) GN=rplA PE=3 SV=1	0.0000	-1.6035
glt1	sp|Q9C102|GLT1_SCHPO Putative glutamate synthase [NADPH] OS=Schizosaccharomyces pombe (strain 972 / ATCC 24843) GN=glt1 PE=2 SV=1	0.0000	-1.6027
glt1	sp|Q9C102|GLT1_SCHPO Putative glutamate synthase [NADPH] OS=Schizosaccharomyces pombe (strain 972 / ATCC 24843) GN=glt1 PE=2 SV=1	0.0000	-1.5711
glt1	sp|Q9C102|GLT1_SCHPO Putative glutamate synthase [NADPH] OS=Schizosaccharomyces pombe (strain 972 / ATCC 24843) GN=glt1 PE=2 SV=1	0.0000	-1.5685
glt1	sp|Q9C102|GLT1_SCHPO Putative glutamate synthase [NADPH] OS=Schizosaccharomyces pombe (strain 972 / ATCC 24843) GN=glt1 PE=2 SV=1	0.0000	-1.5653
MELLADRAFT_90342	tr|F4R8R5|F4R8R5_MELLP Putative uncharacterized protein OS=Melampsora larici-populina (strain 98AG31 / pathotype 3-4-7) GN=MELLADRAFT_90342 PE=4 SV=1	0.0039	-1.5616
fumC	sp|Q9FBN6|FUMC_STRCO Fumarate hydratase class II OS=Streptomyces coelicolor (strain ATCC BAA-471 / A3(2) / M145) GN=fumC PE=3 SV=1	0.0000	-1.5558
fba	sp|Q9X8R6|ALF_STRCO Fructose-bisphosphate aldolase OS=Streptomyces coelicolor (strain ATCC BAA-471 / A3(2) / M145) GN=fba PE=3 SV=1	0.0000	-1.5496
NA	uncharacterized RNA	0.0000	-1.5400

WCM_04769	tr|L2UUH0|L2UUH0_ECOLX Uncharacterized protein (Fragment) OS=Escherichia coli KTE10 GN=WCM_04769 PE=4 SV=1	0.0006	-1.5322
TSTA_040370	tr|B8MI73|B8MI73_TALSN Putative uncharacterized protein OS=Talaromyces stipitatus (strain ATCC 10500 / CBS 375.48 / QM 6759 / NRRL 1006) GN=TSTA_040370 PE=4 SV=1	0.0039	-1.5322
fabG	sp|P71534|FABG_MYCS2 3-oxoacyl-[acyl-carrier-protein] reductase FabG OS=Mycobacterium smegmatis (strain ATCC 700084 / mc(2)155) GN=fabG PE=3 SV=2	0.0000	-1.5095
aceE	sp|Q89AR0|ODP1_BUCBP Pyruvate dehydrogenase E1 component OS=Buchnera aphidicola subsp. Baizongia pistaciae (strain Bp) GN=aceE PE=3 SV=1	0.0000	-1.5053
rpoA	sp|A9WSR1|RPOA_RENSM DNA-directed RNA polymerase subunit alpha OS=Renibacterium salmoninarum (strain ATCC 33209 / DSM 20767 / JCM 11484 / NBRC 15589 / NCIMB 2235) GN=rpoA PE=3 SV=1	0.0000	-1.4797
thrS	sp|A0JXC7|SYT_ARTS2 Threonine--tRNA ligase OS=Arthrobacter sp. (strain FB24) GN=thrS PE=3 SV=1	0.0000	-1.4769
purL	sp|A9WV65|PURL_RENSM Phosphoribosylformylglycinamidine synthase subunit PurL OS=Renibacterium salmoninarum (strain ATCC 33209 / DSM 20767 / JCM 11484 / NBRC 15589 / NCIMB 2235) GN=purL PE=3 SV=1	0.0000	-1.4637
gabT	sp|P40829|GABT_MYCLE 4-aminobutyrate aminotransferase OS=Mycobacterium leprae (strain TN) GN=gabT PE=3 SV=1	0.0000	-1.4481
gluA	sp|P48243|GLUA_CORGL Glutamate transport ATP-binding protein GluA OS=Corynebacterium glutamicum (strain ATCC 13032 / DSM 20300 / JCM 1318 / LMG 3730 / NCIMB 10025) GN=gluA PE=3 SV=2	0.0000	-1.4319
gsiB	sp|Q0TJL8|GSIB_ECOL5 Glutathione-binding protein GsiB OS=Escherichia coli O6:K15:H31 (strain 536 / UPEC) GN=gsiB PE=3 SV=2	0.0000	-1.4266
pnp	sp|Q82K80|PNP_STRAW Polyribonucleotide nucleotidyltransferase OS=Streptomyces avermitilis (strain ATCC 31267 / DSM 46492 / JCM 5070 / NCIMB 12804 / NRRL 8165 / MA-4680) GN=pnp PE=3 SV=1	0.0000	-1.4232
thrS	sp|A0JXC7|SYT_ARTS2 Threonine--tRNA ligase OS=Arthrobacter sp. (strain FB24) GN=thrS PE=3 SV=1	0.0000	-1.4032
tri1	sp|Q9RDE2|TRI1_STRCO Tricorn protease homolog 1 OS=Streptomyces coelicolor (strain ATCC BAA-471 / A3(2) / M145) GN=tri1 PE=1 SV=1	0.0000	-1.3979
aspC	sp|Q9X0Y2|AAT_THEMA Aspartate aminotransferase OS=Thermotoga maritima (strain ATCC 43589 / MSB8 / DSM 3109 / JCM 10099) GN=aspC PE=1 SV=1	0.0000	-1.3886
Namu_4940	tr|C8XA14|C8XA14_NAKMY Rhodanese domain protein OS=Nakamurella multipartita (strain ATCC 700099 / DSM 44233 / JCM 9543 / Y-104) GN=Namu_4940 PE=4 SV=1	0.0000	-1.3597
motA	sp|Q44902|MOTA_BORBU Motility protein A OS=Borrelia burgdorferi (strain ATCC 35210 / B31 / CIP 102532 / DSM 4680) GN=motA PE=3 SV=2	0.0000	-1.3407

fba	sp|Q9X8R6|ALF_STRCO Fructose-bisphosphate aldolase OS=Streptomyces coelicolor (strain ATCC BAA-471 / A3(2) / M145) GN=fba PE=3 SV=1	0.0000	-1.3254
fabH	sp|B2GFZ7|FABH_KOCRD 3-oxoacyl-[acyl-carrier-protein] synthase 3 OS=Kocuria rhizophila (strain ATCC 9341 / DSM 348 / NBRC 103217 / DC2201) GN=fabH PE=3 SV=1	0.0000	-1.3238
infB	sp|A1T7H8|IF2_MYCVP Translation initiation factor IF-2 OS=Mycobacterium vanbaalenii (strain DSM 7251 / PYR-1) GN=infB PE=3 SV=1	0.0000	-1.3022
dnaK	sp|C5C3P2|DNAK_BEUC1 Chaperone protein DnaK OS=Beutenbergia cavernae (strain ATCC BAA-8 / DSM 12333 / NBRC 16432) GN=dnaK PE=3 SV=1	0.0016	-1.2784
hisA	sp|A9WQA3|HIS4_RENSM 1-(5-phosphoribosyl)-5-[(5-phosphoribosylamino)methylideneamino] imidazole-4-carboxamide isomerase OS=Renibacterium salmoninarum (strain ATCC 33209 / DSM 20767 / JCM 11484 / NBRC 15589 / NCIMB 2235) GN=hisA PE=3 SV=1	0.0000	-1.2676
ftsZ	sp|P45499|FTSZ_KOCRD Cell division protein FtsZ OS=Kocuria rhizophila (strain ATCC 9341 / DSM 348 / NBRC 103217 / DC2201) GN=ftsZ PE=3 SV=2	0.0000	-1.2540
kgd	sp|Q1B4V6|KGD_MYCSS Multifunctional 2-oxoglutarate metabolism enzyme OS=Mycobacterium sp. (strain MCS) GN=kgd PE=3 SV=1	0.0000	-1.2438
infB	sp|A1T7H8|IF2_MYCVP Translation initiation factor IF-2 OS=Mycobacterium vanbaalenii (strain DSM 7251 / PYR-1) GN=infB PE=3 SV=1	0.0000	-1.2270
SAV_4896	sp|Q82DS2|Y4896_STRAW UPF0234 protein SAV_4896 OS=Streptomyces avermitilis (strain ATCC 31267 / DSM 46492 / JCM 5070 / NCIMB 12804 / NRRL 8165 / MA-4680) GN=SAV_4896 PE=3 SV=1	0.0000	-1.2061
topA	sp|Q9X909|TOP1_STRCO DNA topoisomerase 1 OS=Streptomyces coelicolor (strain ATCC BAA-471 / A3(2) / M145) GN=topA PE=3 SV=1	0.0000	-1.1939
obg	sp|A0JXJ8|OBG_ARTS2 GTPase Obg OS=Arthrobacter sp. (strain FB24) GN=obg PE=3 SV=1	0.0000	-1.1911
purH	sp|A1SMP8|PUR9_NOCSJ Bifunctional purine biosynthesis protein PurH OS=Nocardioides sp. (strain BAA-499 / JS614) GN=purH PE=3 SV=1	0.0000	-1.1897
Achl_1089	tr|B8HE47|B8HE47_ARTCA Type III restriction protein res subunit OS=Arthrobacter chlorophenolicus (strain ATCC 700700 / DSM 12829 / JCM 12360 / NCIMB 13794 / A6) GN=Achl_1089 PE=4 SV=1	0.0006	-1.1841
eno	sp|B2GM13|ENO_KOCRD Enolase OS=Kocuria rhizophila (strain ATCC 9341 / DSM 348 / NBRC 103217 / DC2201) GN=eno PE=3 SV=1	0.0000	-1.1834
garA	sp|A0QYG2|GARA_MYCS2 Glycogen accumulation regulator GarA OS=Mycobacterium smegmatis (strain ATCC 700084 / mc(2)155) GN=garA PE=1 SV=2	0.0001	-1.1825

glnA	sp|P15106|GLNA_STRCO Glutamine synthetase OS=Streptomyces coelicolor (strain ATCC BAA-471 / A3(2) / M145) GN=glnA PE=2 SV=1	0.0000	-1.1749
yrkF	sp|P54433|YRKF_BACSU UPF0033 protein YrkF OS=Bacillus subtilis (strain 168) GN=yrkF PE=3 SV=1	0.0000	-1.1582
infB	sp|A9WPV8|IF2_RENSM Translation initiation factor IF-2 OS=Renibacterium salmoninarum (strain ATCC 33209 / DSM 20767 / JCM 11484 / NBRC 15589 / NCIMB 2235) GN=infB PE=3 SV=1	0.0001	-1.1424
carB	sp|Q9KXR6|CARB_STRCO Carbamoyl-phosphate synthase large chain OS=Streptomyces coelicolor (strain ATCC BAA-471 / A3(2) / M145) GN=carB PE=3 SV=1	0.0000	-1.1394
Asphe3_18340	tr|F0MBQ7|F0MBQ7_ARTPP Methylmalonyl-CoA epimerase OS=Arthrobacter phenanthrenivorans (strain DSM 18606 / JCM 16027 / LMG 23796 / Sphe3) GN=Asphe3_18340 PE=4 SV=1	0.0000	-1.1362
BRPE67_ACDS23800	tr|A0A060P735|A0A060P735_9BURK Putative membrane protein OS=Burkholderia sp. RPE67 GN=BRPE67_ACDS23800 PE=4 SV=1	0.0047	-1.1130
alaS	sp|A1R709|SYA_ARTAT Alanine--tRNA ligase OS=Arthrobacter aurescens (strain TC1) GN=alaS PE=3 SV=2	0.0000	-1.1086
carB	sp|Q9KXR6|CARB_STRCO Carbamoyl-phosphate synthase large chain OS=Streptomyces coelicolor (strain ATCC BAA-471 / A3(2) / M145) GN=carB PE=3 SV=1	0.0000	-1.1040
Achl_2575	tr|B8HCC4|B8HCC4_ARTCA Uncharacterized protein OS=Arthrobacter chlorophenolicus (strain ATCC 700700 / DSM 12829 / JCM 12360 / NCIMB 13794 / A6) GN=Achl_2575 PE=4 SV=1	0.0001	-1.0962
SSHG_00496	tr|D6B7C1|D6B7C1_9ACTO Retrotransposon protein OS=Streptomyces albus J1074 GN=SSHG_00496 PE=4 SV=1	0.0000	-1.0914
ftsZ	sp|P45499|FTSZ_KOCRD Cell division protein FtsZ OS=Kocuria rhizophila (strain ATCC 9341 / DSM 348 / NBRC 103217 / DC2201) GN=ftsZ PE=3 SV=2	0.0000	-1.0836
Arth_1427	tr|A0JUV1|A0JUV1_ARTS2 HNH endonuclease OS=Arthrobacter sp. (strain FB24) GN=Arth_1427 PE=4 SV=1	0.0000	-1.0767
infB	sp|Q0I7K2|IF2_SYNS3 Translation initiation factor IF-2 OS=Synechococcus sp. (strain CC9311) GN=infB PE=3 SV=1	0.0000	-1.0691
cin	sp|P21703|CIN_BPP7 DNA-invertase OS=Enterobacteria phage P7 GN=cin PE=3 SV=1	0.0000	-1.0686
alaS	sp|A1R709|SYA_ARTAT Alanine--tRNA ligase OS=Arthrobacter aurescens (strain TC1) GN=alaS PE=3 SV=2	0.0000	-1.0672
nocP	sp|P35116|NOCP_AGRT5 Nopaline permease ATP-binding protein P OS=Agrobacterium tumefaciens (strain C58 / ATCC 33970) GN=nocP PE=3 SV=1	0.0000	-1.0522
purH	sp|A1SMP8|PUR9_NOCSJ Bifunctional purine biosynthesis protein PurH OS=Nocardioides sp. (strain BAA-499 / JS614) GN=purH PE=3 SV=1	0.0000	-1.0142
bcp	sp|Q9ZHF0|BCP_BUCAP Putative peroxiredoxin bcp OS=Buchnera aphidicola subsp. Schizaphis graminum (strain Sg) GN=bcp PE=3 SV=2	0.0000	-1.0002

fadA	sp|Q5E8X7|FADA_VIBF1 3-ketoacyl-CoA thiolase OS=Vibrio fischeri (strain ATCC 700601 / ES114) GN=fadA PE=3 SV=2	0.0000	-0.9922
SPCC965.06	sp|O59826|KCAB_SCHPO Putative voltage-gated potassium channel subunit beta OS=Schizosaccharomyces pombe (strain 972 / ATCC 24843) GN=SPCC965.06 PE=3 SV=1	0.0000	-0.9920
