# Supplementary material for: Transcriptomic Reprograming of Xanthomonas campestris pv. campestris after Treatment with Hydrolytic Products Derived from Glucosinolates
Source: Plants (Basel). 2021 Aug 11;10(8):1656. doi: 10.3390/plants10081656 (PMC8400333; doi:10.3390/plants10081656)
Supplement: Supplementary file 1 [file plants-10-01656-s001.zip › supplementary/Table S3.pdf]

Table S3. Primers used for RT-qPCR

Gene	Forward primer	Left primer
Cell division protein (ftsX)	atcgaccaccatctgcaca	agattgatttcgcgcgactg
Chaperone protein (dnaK)	gtgaccaacccgaagaacac	tcggcggtcttcttcatctt
Chromosomal replication initiator protein (dnaA)	acacctttgccaacttcgtg	gctgaagaactgttccgagc
Regulatory protein (recX)	ctggaccgaaaacgcactc	ctcaaggtcgaagcgtgttg
Periplasmic trehalase (treA)	cgcgaggtgtattactggga	tcaggtagtaggtgcggttg
Enolase (eno)	ttccattcgctgaagtcggt	ccaggttgtacttgccgttg
Acetyl-coenzyme A synthetase (acsA)	aggacgacatctactggtgc	aagatggtgacctggtgctt
Succinate dehydrogenase flavoprotein subunit (sdhA)	ggacctgatcgagacctacg	gtgaccagtgtgtgcttctg
ATP synthase subunit alpha (atpA)	cgaatacttcatggaccgcg	cttctccacgtactcctcgg
LPS-assembly protein (lptD)	agcagaccatcgaacaagga	caatgaggttgcgacggtag
Protein translocase (secD)	aacagcatccaggtctcgtt	cagtgcattgacccggttac
Glutathione transferase (gst1)	tacatgttccccaagcggat	atccaactccgggtaatgcc
DNA gyrase subunit B (gyrB)	taccaccgcatcatcctg	gctgtatctcaaggac
